# Endothelial cell infection and dysfunction, immune activation in severe COVID-19

**DOI:** 10.7150/thno.61810

**Published:** 2021-07-06

**Authors:** Zhongnan Qin, Fengming Liu, Robert Blair, Chenxiao Wang, Haoran Yang, Joseph Mudd, Joshua M Currey, Naoki Iwanaga, Jibao He, Ren Mi, Kun Han, Cecily C. Midkiff, Mohammad Afaque Alam, Bertal H Aktas, Richard S Vander Heide, Ronald Veazey, Giovanni Piedimonte, Nicholas J Maness, Süleyman Ergün, Franck Mauvais-Jarvis, Jay Rappaport, Jay K. Kolls, Xuebin Qin

**Affiliations:** 1 Tulane National Primate Research Center, Covington, LA 70433, USA; 2 Departments of Medicine and Pediatrics, Center for Translational Research in Infection and Inflammation, Tulane University School of Medicine, New Orleans, LA 70112, USA; 3 Coordinated Instrumentation Facility, Tulane University, New Orleans LA 70118, USA; 4 Department of Medicine, Brigham and Women's Hospital and Harvard Medical School, Boston, MA, USA; 5 Department of Pathology, LSU Health Sciences Center, New Orleans, LA 70112, USA; 6 Departments of Pediatrics, Biochemistry & Molecular Biology, Tulane University School of Medicine, New Orleans, LA 70112, USA; 7 Department of Immunology and Microbiology, Tulane University School of Medicine, New Orleans, LA 70112, USA; 8 Institute of Anatomy and Cell Biology, Julius-Maximilians-Universität Würzburg, Koellikerstrasse 6, 97070 Würzburg, Germany; 9 Department of Medicine, Section of Endocrinology and Metabolism, Tulane University Health Sciences Center, School of Medicine, New Orleans, LA 70112, USA; 10 Southeast Louisiana Veterans Health Care System, New Orleans, LA 70119, USA; 11 Tulane Center of Excellence in Sex-Based Biology & Medicine, LA 70112, USA

## Abstract

**Rationale**: Pulmonary vascular endotheliitis, perivascular inflammation, and immune activation are observed in COVID-19 patients. While the initial SARS-CoV-2 infection mainly infects lung epithelial cells, whether it also infects endothelial cells (ECs) and to what extent SARS-CoV-2-mediated pulmonary vascular endotheliitis is associated with immune activation remain to be determined.

**Methods**: To address these questions, we studied SARS-CoV-2-infected *K18-hACE2* (*K18*) mice, a severe COVID-19 mouse model, as well as lung samples from SARS-CoV-2-infected nonhuman primates (NHP) and patient deceased from COVID-19. We used immunostaining, RNAscope, and electron microscopy to analyze the organs collected from animals and patient. We conducted bulk and single cell (sc) RNA-seq analyses, and cytokine profiling of lungs or serum of the severe COVID-19 mice.

**Results**: We show that SARS-CoV-2-infected *K18* mice develop severe COVID-19, including progressive body weight loss and fatality at 7 days, severe lung interstitial inflammation, edema, hemorrhage, perivascular inflammation, systemic lymphocytopenia, and eosinopenia. Body weight loss in *K18* mice correlated with the severity of pneumonia, but not with brain infection. We also observed endothelial activation and dysfunction in pulmonary vessels evidenced by the up-regulation of VCAM1 and ICAM1 and the downregulation of VE-cadherin. We detected SARS-CoV-2 in capillary ECs, activation and adhesion of platelets and immune cells to the vascular wall of the alveolar septa, and increased complement deposition in the lungs, in both COVID-19-murine and NHP models. We also revealed that pathways of coagulation, complement, K-ras signaling, and genes of ICAM1 and VCAM1 related to EC dysfunction and injury were upregulated, and were associated with massive immune activation in the lung and circulation.

**Conclusion**: Together, our results indicate that SARS-CoV-2 causes endotheliitis via both infection and infection-mediated immune activation, which may contribute to the pathogenesis of severe COVID-19 disease.

## Introduction

SARS-CoV-2 has killed more than one million people globally. However, we have not yet developed highly effective therapeutics for treatment of the COVID-19 disease 1. This is partially due to a limited understanding of COVID-19 pathogenesis. COVID-19 patients display a wide range of symptoms including severe illness associated with disseminated platelet activation and coagulation, cytokine storm, multiple organ failure, and death. The presence of widespread microthrombi and endothelial cell (EC) damage throughout the pulmonary vasculature suggests that vasculopathy is important in COVID-19 pathogenesis 2, 3. A better understanding of these pathogenic mechanisms offers important opportunities to identify novel agents or repurpose existing drugs to attenuate viral attachment and replication, inflammation, EC damage, blood clotting and systemic microangiopathy 4, 5.

SARS-CoV-2 primarily infects pneumocytes expressing the SARS-CoV-2 receptor ACE2, which facilitates viral entry. Little is known about SARS-CoV-2-mediated direct or indirect vasculopathy and its possible role in disease outcomes 6, 7. Studies indicate that in a variety of tissues, and especially in the lung, ECs may be infected by the virus 2, 8-11, although they express lower ACE2 levels compared to pneumocytes 1. Direct EC infection may explain many clinical features of advanced disease, including disseminated activation of coagulation pathways and platelets and propensity to thrombosis, especially in the lung. It has been hypothesized that COVID-19-related acute respiratory distress syndrome (ARDS) is a vascular endotype or even an EC disease 12, 13. However, whether SARS-CoV-2 directly infects microvascular EC in organs, including the lung, remains uncertain 14. In addition, the identification of SARS-CoV-2 in human EC by electron microscopy (EM) has been demonstrated but remains a subject of scientific debate 11, 15-17. The direct infection of EC by SARS-CoV-2 in the lung and other organs have not been investigated experimentally 1. Further, using the COVID-19 rhesus macaques' model, previous study indicates that SARS-CoV-2-mediated indirect endothelial disruption and vascular thrombosis via interactions between immune and inflammatory pathways contribute to SARS-CoV-2-induced EC dysfunction 18. However, rhesus macaques do not develop severe COVID-19, and thus cannot provide information on the pathogenesis of severe disease in humans 18. Also, most of clinical studies showing EC infection and dysfunction were conducted on autopsied human samples, which makes it difficult to identify the pathogenic role of EC infection and dysfunction and the underlying mechanisms of alterations observed. Together, the relative contribution of SARS-CoV-2-mediated direct EC infection and indirect immune-mediated EC dysfunction in COVID-19 still requires investigation 5.

To address these questions, we used a mouse model of severe COVID-19, SARS-CoV-2-infected *K18-hACE2* (*K18*) mice, as well as tissue from SARS-CoV-2-infected NHPs and samples from autopsy of COVID-19 patient. We show that, in addition to infecting pneumocytes, SARS-CoV-2 also infects EC. We observe that SARS-CoV-2-mediated EC dysfunction is associated with immune, complement and platelet activation/thrombosis in the lung of the *K18* mice and NHPs. Our results suggest that SARS-CoV-2-mediated direct EC infection and indirect EC dysfunction may contribute to the pathogenesis of COVID-19. These results also indicate that pharmacologic suppression of EC infection and dysfunction may have a beneficial effect in treating or reducing the severity of COVID-19 and possibly diseases associated with emerging coronaviruses.

## Results

### SARS-CoV-2-infected *K18* mice developed severe COVID-19

**Figure 1:**
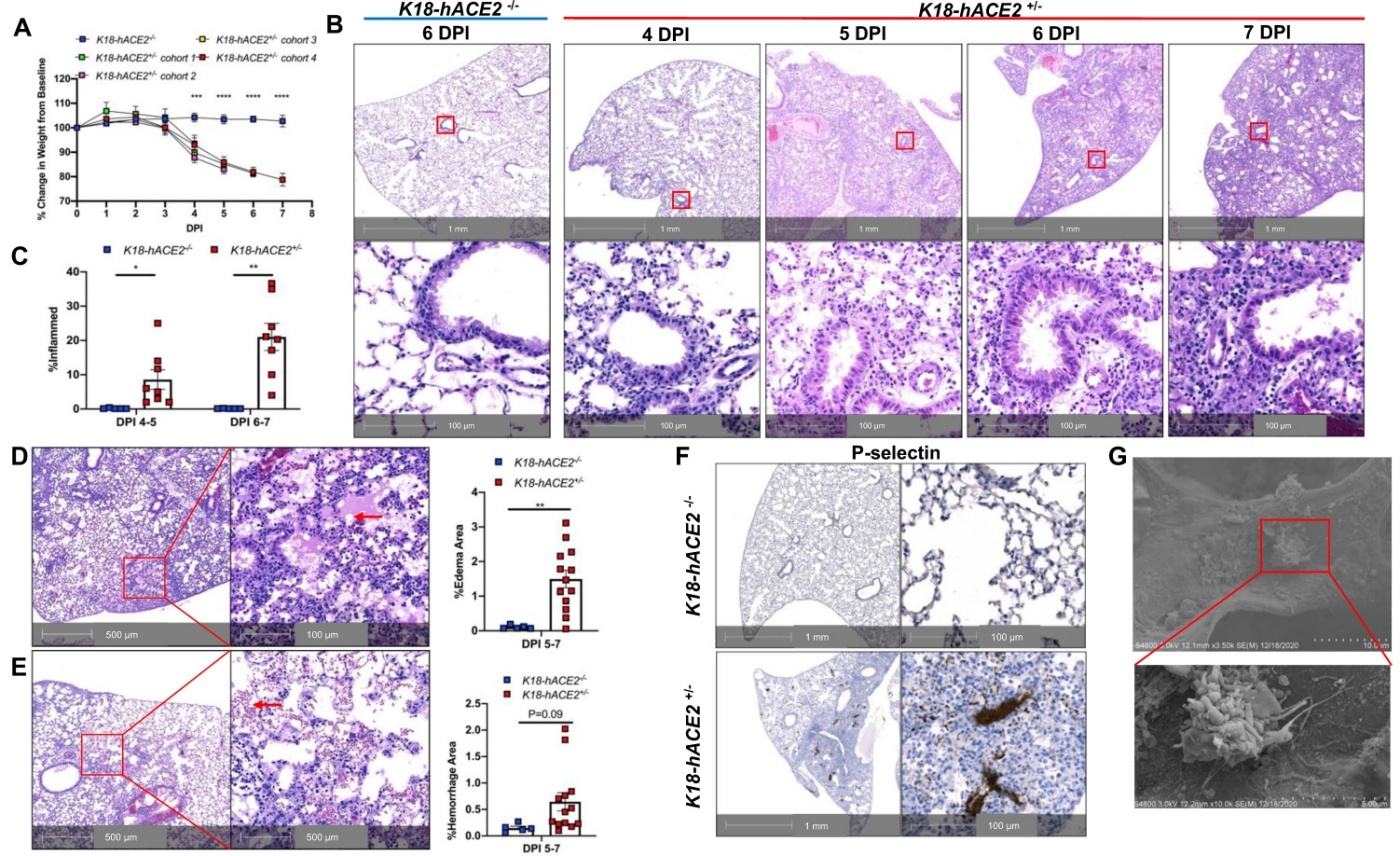
** SARS-CoV-2-infected *K18* mice develop severe COVID-19 disease. (A)** Male and female *K18*^-/-^ and 4 cohorts of *K18*^+/-^ were infected with 2 x10^5^ TCID of SARS-CoV-2 intranasally and body weight change was monitored. *** P < 0.001; **** P < 0.0001 (*K18*^+/-^ Cohort 4 vs. *K18*^-/-^ group). (n = 5 for cohort 2,3,4; n = 3 for cohort 1; n = 5 for *K18*^-/-^ mice). **(B)** Representative H&E staining of lung sections from infected *K18*^-/-^ at 6 DPI and *K18*^+/-^ at 4, 5, 6 and 7 DPI. **(C)** Quantification of lung inflammation by percentage of the lung affected at 4-5 and 6-7 DPI. *P < 0.05, **P < 0.01. Analyzed by unpaired Student's T-test (n = 5 for *K18*^-/-^; n = 8 for *K18*^+/-^ at each time point) **(D-E)** Representative H&E staining of lung sections from *K18*^+/-^ at 5 and 6 DPI shows (D) extensive edema (red arrows), and (E) pulmonary hemorrhage indicated by red blood cells in alveolar spaces (red arrows). Quantification of edema and hemorrhage by percentage of the lung affected at 5-7 DPI. (n = 5 for *K18*^-/-^; n = 13 for *K18*^+/-^. Analyzed by unpaired student t-test, **P < 0.01)** (F)** Representative immunohistochemistry staining of P-selectin in lung sections from *K18*^-/-^ and *K18*^+/-^ at 6 DPI. **(G)** Scanning EM images show small thrombosis on pulmonary capillary vessel wall of *K18*^+/-^ at 5 DPI. All data are presented as mean ± SEM.

To investigate the potential roles of immune activation, EC infection, and dysfunction underlying severe COVID-19, we established and characterized SARS-CoV-2-infected *K18* mice. *K18* transgenic mice express hACE2 in airway epithelial cells under the control of the human cytokeratin 18 (*K18*) promoter 19. We infected *K18* mice with a moderate dose of SARS-CoV-2 (2 X 10^5^ TCID50) and euthanized the mice at 4-, 5-, 6-, and 7-days post infection (DPI). Consistent with previous findings 20-24, we found that SARS-CoV-2 infection of *K18* mice induced a progressive body weight loss from 4 to 7 DPI and fatality at 7 DPI, compared with infected non-transgenic wild type (WT) mice (**Figure 1A**). The mice developed mild to severe interstitial pneumonia with progressive perivascular inflammation (**Figure 1B-C**), alveolar edema (**Figure 1D**), and hemorrhage (**Figure 1E**) at 4 to 7 DPI. Of note, these mice developed pathological changes with various degrees of edema, hemorrhage, or inflammation at different time intervals (**Figure 1B-E**), and these histological changes were similar to those seen in severe COVID-19 patients 25, 26. After 5 DPI, mice exhibited at least one of these histological changes, all of which contribute to the severe COVID-19 phenotype. Further, in addition to the lung histopathology described above, *K18* mice euthanized at 6 DPI also exhibited some degree of the pulmonary thrombosis (**Figure 1F**). We then stained the lung sections for P-selectin and CD41, well established biomarkers of platelet activation and aggregation 27-29. Severe to mild pulmonary platelet activation was observed by P-selectin (**Figure 1F** and **Figure S1A-D**) and CD41 staining of the lungs of the infected *K18* mice (**Figure S2**).

Additionally, infected *K18^+/-^* mice had fewer white blood cells, including lymphocytes and eosinopenia compared to infected WT (or *K18^-/-^*) mice at either 4 or 6 DPI (**Table S1**). Reduction in these cell counts has been linked to severe COVID-19 in humans 30, 31. Serum chemistry analysis showed increased levels of LDH, a routinely used clinical biomarker for organ damage 32, as well as AST and ALT, indicative of liver injury (**Figure S3**). Such changes in serum chemistry analytes suggest involvement of other organs, especially the liver. We interpreted the decrease in blood glucose and triglyceride seen in infected-*K18* mice compared to controls as the consequence of body weight loss in these mice (**Figure S3**).

To examine SARS-CoV-2 infection in tissues of *K18* mice, we used qRT-PCR to quantify the nucleocapsid (N) sub-genomic viral RNA, a correlate of active virus replication 33, 34, in organs daily until 6 DPI (**Figure S4**). We found that the lungs of SARS-CoV-2-infected *K18* mice had, on average, 10^5^ (at 1 DPI), 10^9^ (at 2 and 3 DPI), and 10^6^ (at 4- to 6- DPI) sub-genomic N RNA copies per 100ng of tissue RNA. This dynamic growth of replicating virus was further confirmed by direct viral staining, a reliable and sensitive method for detecting replicating virus in tissues 34 (**Figure S5A-B**). Further studies revealed that the brains of infected *K18* mice had 10^3^ (at 1, 2, 3 DPI), and 10^8^ (at 4-6 DPI) subgenomic viral RNA load (**Figure S4**), suggesting that the lung had been infected earlier than the brain. In addition, SARS-CoV-2-infected *K18* mice exhibited several logs higher genomic viral load in the liver but not in the spleen, compared to the infected nontransgenic littermate (or WT) mice (**Figure S4**). It is notable that viral loads of infected *K18* mice were much higher in lung and brain than in spleen and liver (**Figure S4**).

Together, these findings indicate that *K18* mice infected with a moderate dose of SARS-CoV-2 primarily results in infection of the lung and brain. The mice developed severe COVID-19 associated with severe lung histological changes.

### Lung disease is the primary determinant of COVID-19 severity in *K18* mice

**Figure 2:**
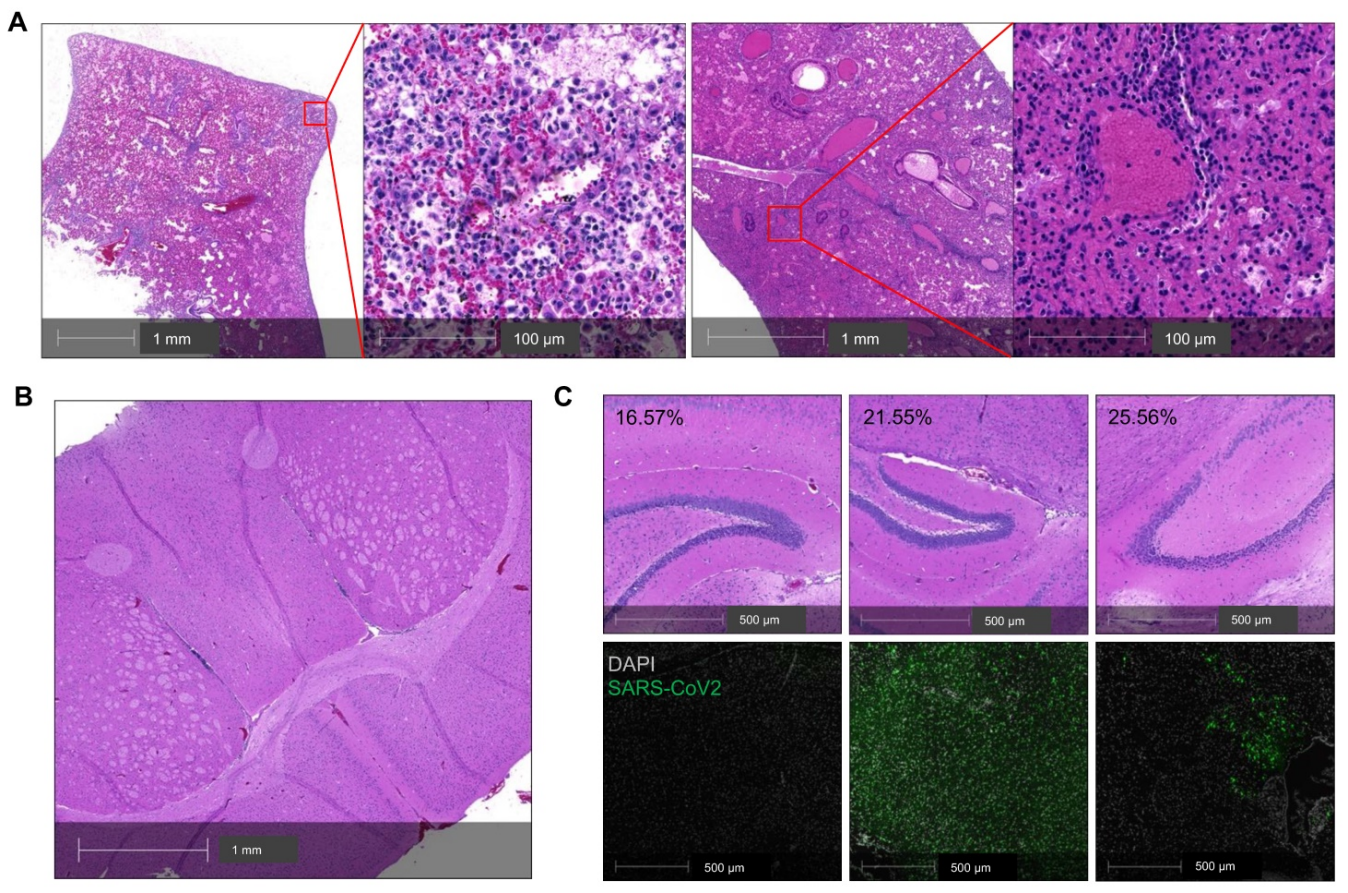
** Lung infection mainly contributes to the severity of COVID-19 in SARS-CoV-2-infected *K18* mice. (A)** Representative H&E staining of lung sections from a *K18*^+/-^ mouse that died at 7 DPI. Left panel shows perivascular infiltration by lymphocytes, edema, hemorrhage, multifocal regions of neutrophilic inflammation; right panel shows a congested vessel. (**B**) Representative H&E section of cerebrum from the postmortem *K18*^+/-^ mouse at 7 DPI showing no overt pathology and mild autolytic changes. **(C)** H&E staining (upper panel) and corresponding immunofluorescence staining of viral spike protein (lower panel, green) in the brain of 3 *K18*^+/-^ at 7 DPI**.** Corresponding %weight loss changes are shown in upper panel.

The infected* K18* mice also developed progressive body weight loss. Whether this body weight loss was related to lung or brain infection remains unclear 35, and this question is relevant to the selection of COVID-19 model for pathogenesis studies. To address this question, we analyzed a cohort of five infected *K18* mice including two mice that died at 7 DPI (**Figure 2A-C**). Histologic analysis revealed moderate postmortem autolysis with pulmonary perivascular infiltration by low to moderate numbers of lymphocytes, widespread edema, multifocal neutrophilic inflammation (**Figure 2A** left panel), and congested blood vessel (**Figure 2A** right panel). We did not observe obvious brain lesions either in this mouse (**Figure 2B**) or the other three mice euthanized at 7 DPI (**Figure 2C**). We excluded another mouse that had died from the analysis due to severe postmortem autolysis. Direct staining of the brain for viral antigens was used to assess the severity of brain infections among these three mice. While the three mice had lost 16.6, 21.6, and 25.6% of their body weight respectively, body weight loss did not correlate with the severity of the infection within the brain (**Figure 2C**). These results suggest that lung infection is the primary determinant of the body weight loss in this mouse model.

We further explored SARS-CoV-2-mediated damage in other organs of the three mice euthanized at 7 DPI and one mouse that died at 7 DPI. We observed organ damage in 3 of 4 mice. One mouse exhibited multiple organ damage including cerebral infarction, minimal myocarditis, minimal splenic congestion and hepatocellular vacuolation (**Figure S6A-D**). Another mouse exhibited minimal myocarditis** (Figure S6E)** and the third mouse exhibited minimal hepatitis (**Figure S6F**). Our results demonstrate that severe COVID-19 in *K18* mice results in damage to organs other than the lung as seen in severe COVID-19 patients, but the lung injury remains the primary determinant of the clinical manifestations of severe COVID-19 including pneumonia, body weight loss, and fatality.

### SARS-CoV-2 infection causes EC activation and dysfunction

**Figure 3:**
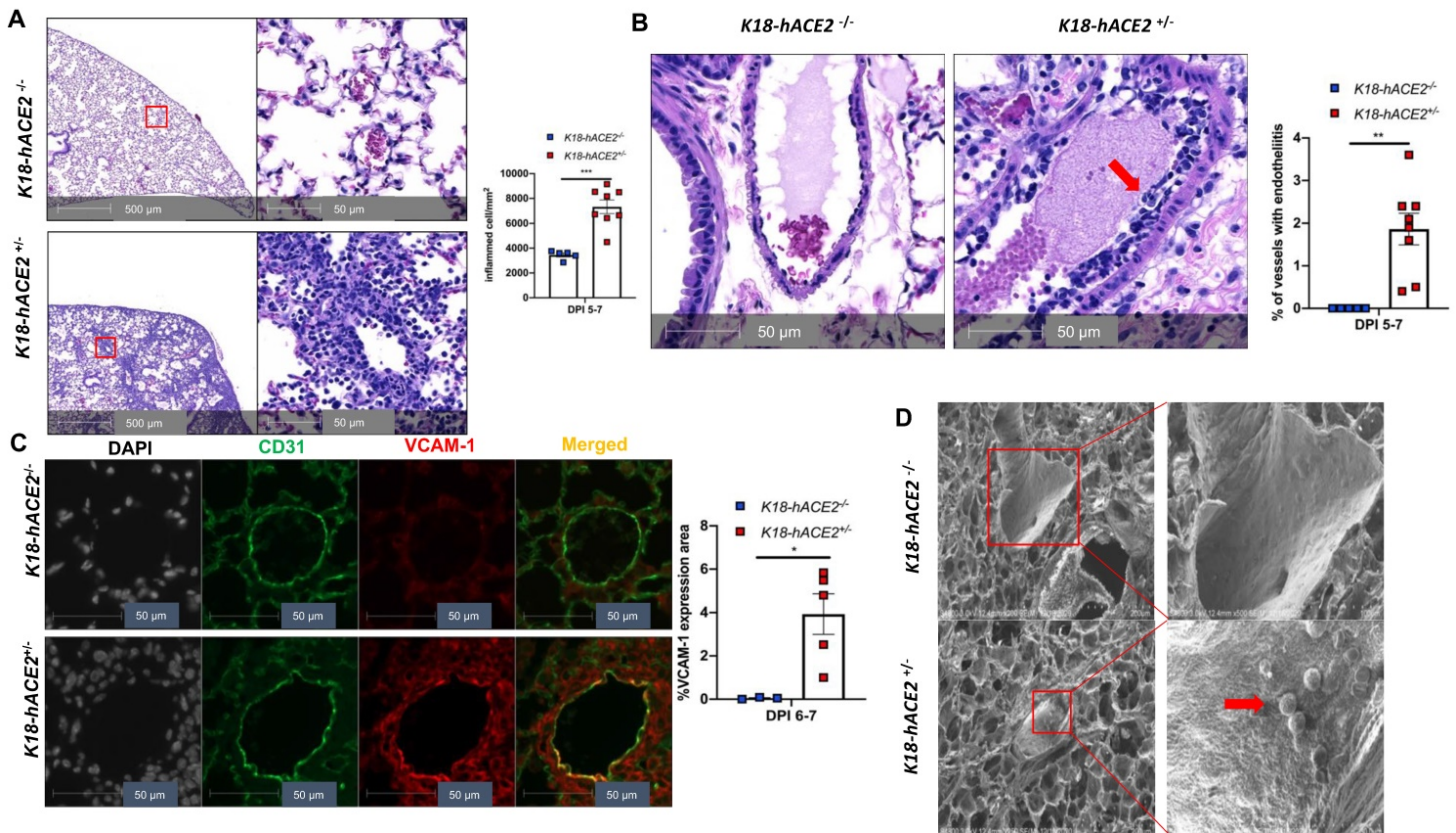

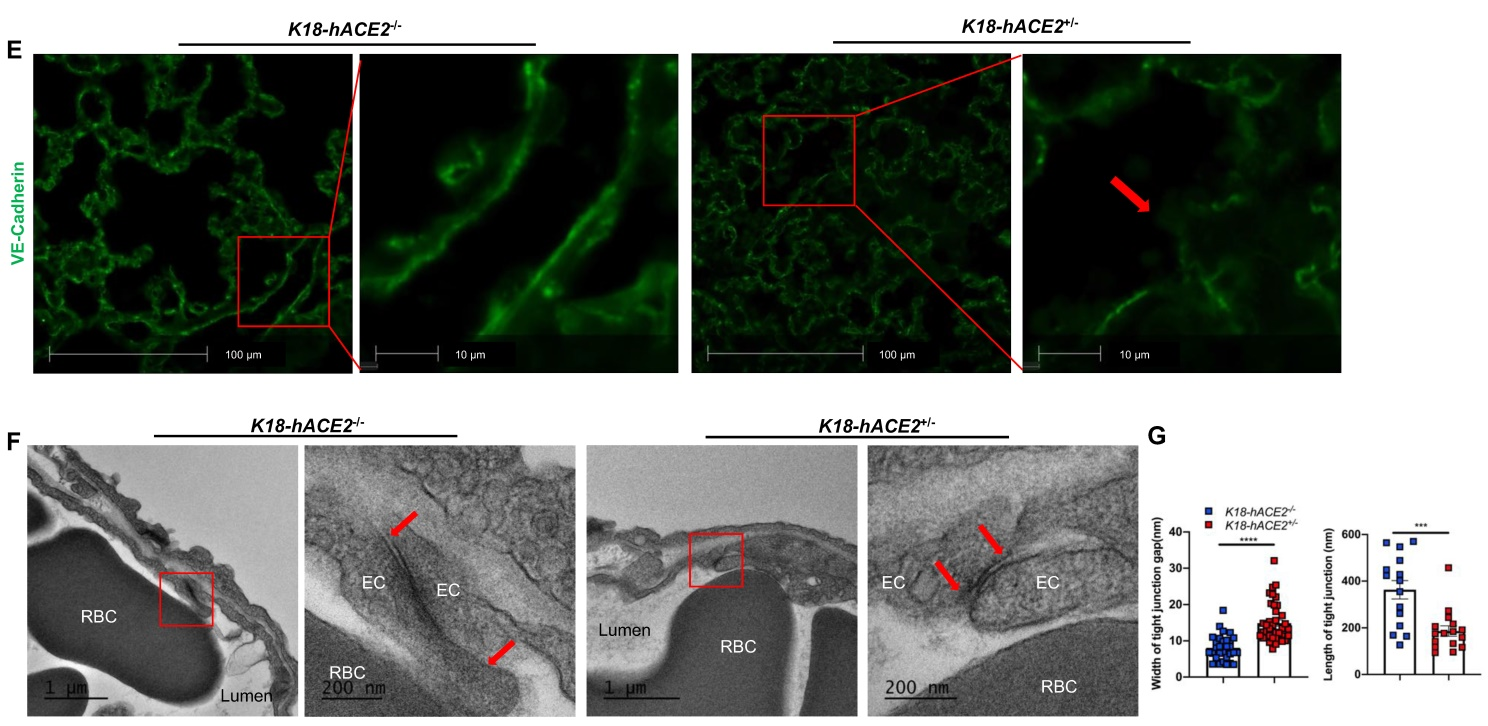
** SARS-CoV-2 infection induces EC activation and dysfunction. (A)** Representative H&E staining shows perivascular inflammation in the lung of *K18*^+/-^, but not *K18*^-/-^ at 5-7 DPI. Quantification of perivascular inflammation by counting inflamed cells around vessels in the lung sections of infected mice at 5-7 DPI (n = 5 for *K18*^-/-^; n = 8 for *K18*^+/-^). **(B)** Endotheliitis of a pulmonary vessel with marked infiltration of the endothelium by inflammatory cells and sloughing of ECs (arrow in middle panel). Quantitative analysis of the percentages of vessel with endotheliitis in the mice (right panel). **(C)** Co-staining of CD31 (green) and VCAM1 (red) in the lung sections from *K18*^-/-^ (upper panel) and *K18*^+/-^ (lower panel) at 6 DPI. Nuclei are stained with DAPI (White). Images are representative of 3-5 mice for each group**.** Quantification of VCAM-1 expressions is presented by the VCAM1 positive area/lung area x100 % (n = 3 for *K18*^-/-^; n = 5 for *K18*^+/-^ at 6-7 DPI). **(D)** Scanning EM images show immune cells came into contact with pulmonary vessel wall of *K18*^+/-^ mice at 5 DPI. **(E)** Immunofluorescence staining of VE-cadherin in the lung sections from infected *K18^-^*^/-^ (left panel) and *K18^+/-^* (right panel) at 6 DPI. Red arrows indicate breakdown of EC barrier. Images are representative of 3 mice for each group**. (F)** Transmission EM images of *K18*^+/-^ show smaller and wider tight junction as compared with *K18*^-/-^ at 5 DPI. RBC: red blood cells; Lumen: vascular lumen** (G)** Quantification of width of individual tight junction gap and length of tight junction at 5 DPI. Data are shown as mean ± SEM. * P < 0.05; ** P < 0.01; *** P < 0.001; **** P < 0.0001, as determined by unpaired two-tailed Student's t-test.

Our experimental results described above suggest that SARS-CoV-2 infection in *K18* mice causes severe COVID-19 phenotype. In addition to the typical lung histological changes described above, perivascular pulmonary inflammation has been reported in many COVID-19 animal models, including nonhuman primates 36, mice 37, hamsters 38, and ferrets 35. We also observed the same perivascular inflammation in our severe COVID-19 mouse model (**Figure 3A**). Endotheliitis has been previously shown in the endothelium and perivascular space of SARS-CoV-2-infected humans and NHPs, and is previously characterized by hyperplastic endothelium, intimal proliferation and adherence of macrophages and lymphocytes 3, 18, 39. Accordingly, we found that SARS-CoV-2 infected *K18* mice also exhibited prominent endotheliitis confirmed by histological studies (**Figure 3B**). The detection of increased intercellular adhesion molecule 1 (ICAM-1) and vascular cell adhesion molecule 1 (VCAM1) by immunostaining is widely used to assess EC activation and dysfunction 40. We found that infected *K18* but not WT mice had dramatically increased co-localization of VCAM1 and ICAM-1 with the EC marker CD31 in the pulmonary vessels (**Figure 3C** and **Figure S7A**), and increased immune cells adherence to pulmonary EC, observed by ultra-structural analysis (**Figure 3D**). Additionally, we examined vascular barrier integrity by staining with VE-cadherin, which plays a central role in permeability changes by controlling the opening and closing of the EC barrier 41, 42. We found decreased expression of VE-cadherin in the pulmonary vasculature (**Figure 3E** and **Figure S7B-C**) and associated increased gap and decreased length of the tight junction in the areas of inter-EC contacts (**Figure 3F-3G**) of *K18* mice, but not that of non-transgenic littermates infected with SARS-CoV-2, which is indicative of EC barrier disruption and thus increased permeability. These results suggest the existence of extensive EC activation, thrombosis and disrupted EC integrity in *K18* mice. Thus, we conclude that SARS-CoV-2 infection in *K18* mice induces EC activation and dysfunction, leading to increased vascular permeability.

### SARS-CoV-2 infects ECs

**Figure 4:**
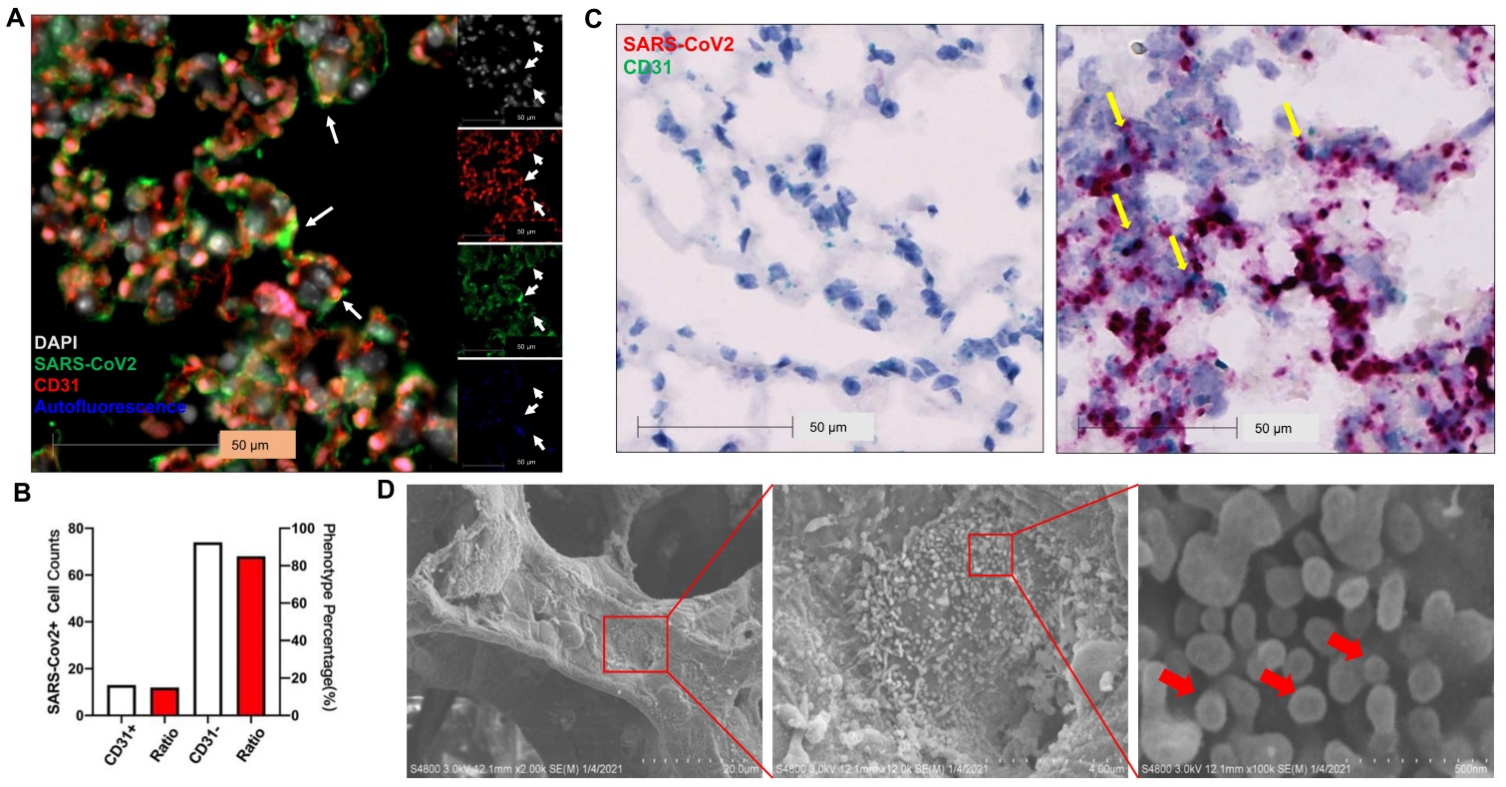
** SARS-CoV-2 Infects ECs. (A)** Representative immunofluorescence staining of CD31 (red) and viral spike protein (green) in lung sections from infected *K18*^+/-^ at 3 DPI. Nuclei are stained with DAPI (white). White arrows: co-localization of CD31 and SARS-CoV-2 protein. Autofluorescence identifies red blood cells which fluoresce in the green (488), red (568), and blue (647) channels. (**B**) Quantification of cell counts (left axis) and percentage of CD31+ and CD31- cells (right axis) of total infected cells.** (C)** In situ hybridization (ISH) staining of SARS-CoV-2 RNA (red) and CD31 RNA (green) in the lung sections from infected *K18^-/-^* (left) and *K18*^+/-^ (right) at 3 DPI. Yellow arrows: co-localization of CD31 (green) and viral RNA (red). Images are representative of 2 mice for each group. **(D)** Scanning EM images of lung sections from infected *K18*^+/-^ mice at 5 DPI shows the shedding viral particles (red arrows) on pulmonary vessel wall.

Previous studies show that SARS-CoV-2 infects pneumocytes in *K18* mice 22, 23 and SARS-CoV-2 N protein was occasionally observed in the EC lining of the pulmonary vessels 23. Whether EC infection occurs in this model remains to be determined. Using immunostaining, we found SARS-CoV-2 co-localized in alveolar septa with CD31, a biomarker for ECs (**Figure 4A**) and 15% of SARS-CoV-2-stained cells co-stained with CD31 in the infected *K18* mice at 3 DPI (**Figure 4B**). Using RNAscope, we also found that SARS-CoV-2 spike RNA co-localized with the CD31 RNA in the alveolar septa (**Figure 4C**). EM also revealed large numbers of SARS-CoV-2 viral particles overlying ECs in alveolar capillaries (**Figure 4D**). These results indicate that ECs can be infected by SARS-CoV-2. We further investigated the kinetics of the EC infection by performing the co-staining of the lungs of the infected *K18* mice at 1 (early course of the disease) and 6 DPI (late course of the disease). We detected the co-staining of SARS-CoV-2 with CD31 at both time intervals although the mice at both times had less viral staining in the lungs compared to the mice at 3 DPI (**Figure S5**). This suggests that SARS-CoV-2 infects ECs in an entire course of the disease and implies that direct EC infection may contribute to the EC dysfunction found in COVID-19. Additionally, we also demonstrated the co-localization of SARS-CoV-2 with hACE2 and serine transmembrane protein 2 (Tmprss2) (**Figure S8**), an established receptor and recently known co-receptor for the viral infection, respectively 1, 43-46. These results further support the notion that transgenic expression of hACE2 in K18 mice sensitizes the mice to the infection and mouse Tmprss2 may also contribute to the infection 1, 43-46.

### SARS-CoV-2-mediated EC activation is also seen in the lungs of SARS-CoV-2-infected NHPs and a human patient

Previous histological analysis revealed the existence of EC barrier disruption and vascular thrombosis in SARS-CoV-2-infected rhesus macaques 18.

**Figure 5:**
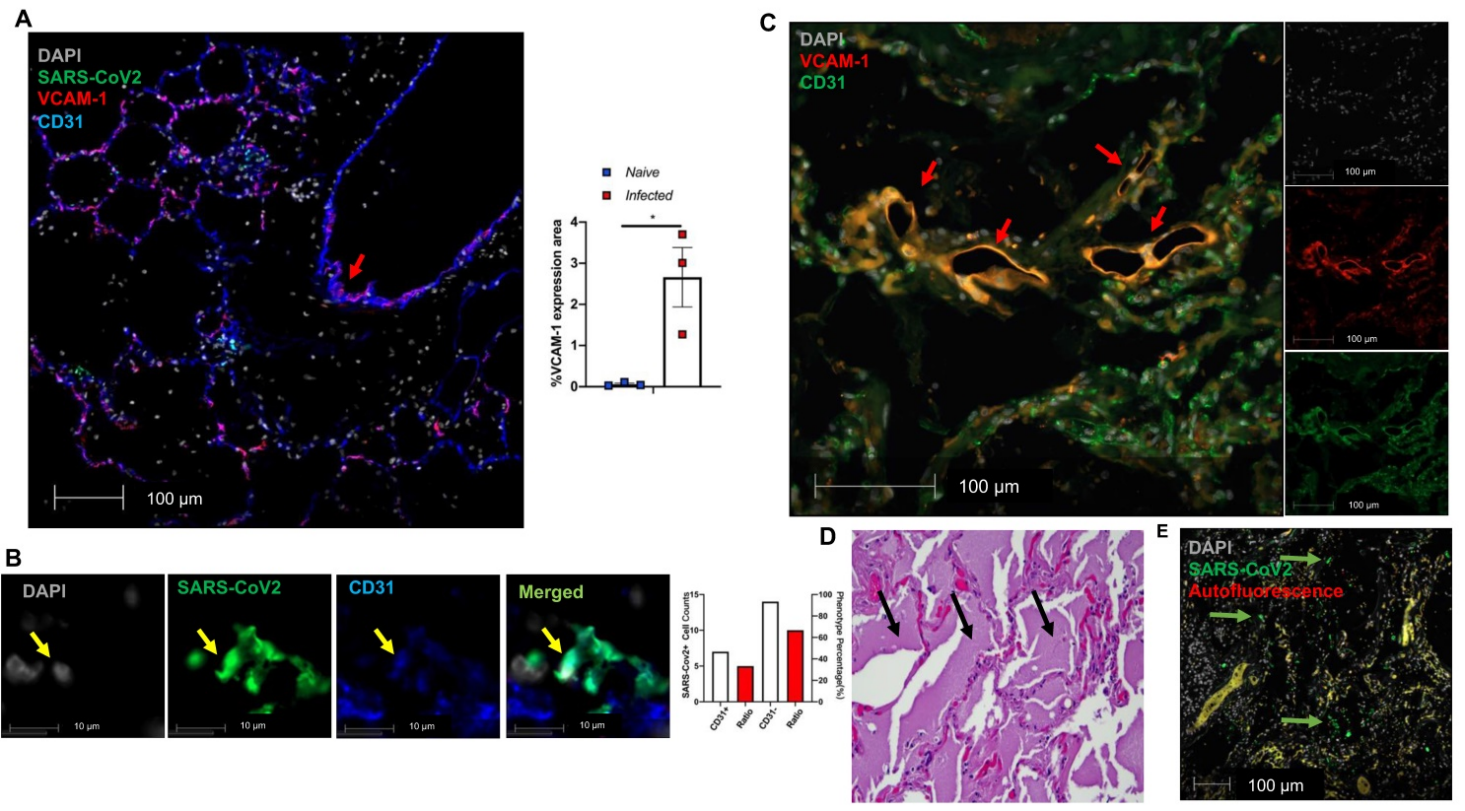
** Extensive EC activation and infection in SARS-CoV-2 infected AGMs and human. (A-B)** Co-staining of viral protein (green, yellow arrow in Fig. 5B), CD31 (blue) and VCAM1 (red) in lung sections from SARS-CoV-2-infected AGMs. (A) Co-localization of VCAM1 and CD31 (red arrow). Graph shows the quantification of VCAM1 expression in SARS-CoV-2-infected and naïve AGMs. The data shown is the VCAM1 positive area/lung area x100 %. (n = 3 for each group). (B) Co-localization of SARS-CoV-2 protein (green) and CD31 (blue); Nuclei are stained with DAPI (white). Quantification of cell counts (left axis) and percentage of CD31+ and CD31- cells (right axis) of total infected cells.** (C-E)** EC dysfunction in an archived lung of autopsied COVID-19 patient**.** The patient was a 78-year-old female with a history of end-stage renal disease, type 2 diabetes and obesity who presented in cardiac arrest after a two-day history of shortness of breath. (C) Lung sections exhibit co-staining (arrows) of VCAM1 (red) and CD31 (green). (D) H&E stains demonstrate abundant, acute, pulmonary edema (black arrows). (E) SARS-CoV-2 positive cells (green) are scattered throughout sections of the lung (green arrows).

We further evaluated EC dysfunction in using our published and archived SARS-CoV-2 infected NHP lungs 47. We also documented extensive VCAM1 expression in the pulmonary vessel of a SARS-CoV-2 infected African green monkey (AGM), NC34, that was euthanized due to ARDS 47 (**Figure 5A,** red arrow in left panel). Other infected AGM (NC33 and NC40) also exhibited significantly higher VCAM1 staining in the lung than naïve monkeys (**Figure 5A** right panel). Both NC34 and NC33 developed ARDS characterized by fulminant respiratory distress, radiographic lung opacity, and diffuse alveolar damage at 22 DPI 47. NC40 survived to the end of the study (26 DPI) and showed mild interstitial pneumonia 47. Consistently, we observed EC infection evidenced by co-localization of SARS-CoV-2 with CD31 within the alveolar septa (**Figure 5B**). We also found EC dysfunction in an autopsied lung collected from a patient who died of severe COVID-19 as evidenced by VCAM1 staining (**Figure 5C**), which is associated with extensive edema (**Figure 5D**), and viral infection evidenced by extensive viral staining in the lung (**Figure 5E**). Together, we also demonstrate EC infection by SARS-CoV-2 and dysfunction mediated by SARS-CoV-2 infection in the lungs of COVID-19 NHP and human samples.

### RNA seq, scRNA seq, lung tissue array, and serum cytokine analyses

**Figure 6:**
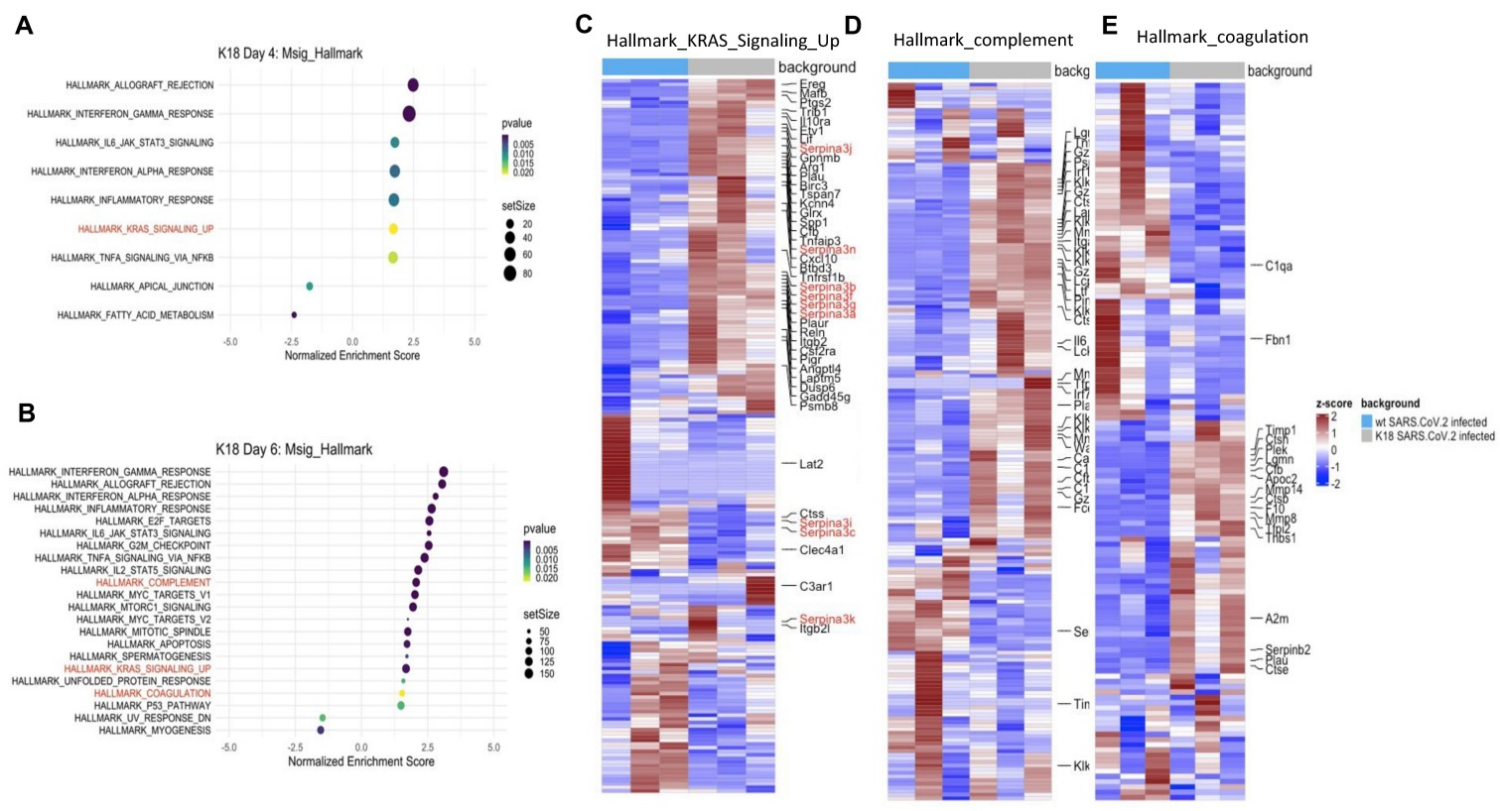
** Lung tissue of SARS-CoV-2-infected *K18* mice reveals gene signatures of EC inflammation.** Transcripts from bulk RNA-seq of lung tissue from WT and *K18* mice infected with SARS-CoV-2 were analyzed by pathway analysis. Enrichment plot of highly enriched biological pathways from the Molecular Signatures Hallmark gene set collection at 4 days post infection (**A**) and 6 days post infection (**B**). Curated heatmaps of (**C**) K-ras signaling, (**D**) complement, and (**E**) coagulation gene sets. Annotated genes are those that were found to be differentially expressed between SARS-CoV-2-infected *K18* and wt groups. Significance in (**A**) was determined by a Fisher's exact test (panel). All data are presented as mean ± SEM.

**Figure 7:**
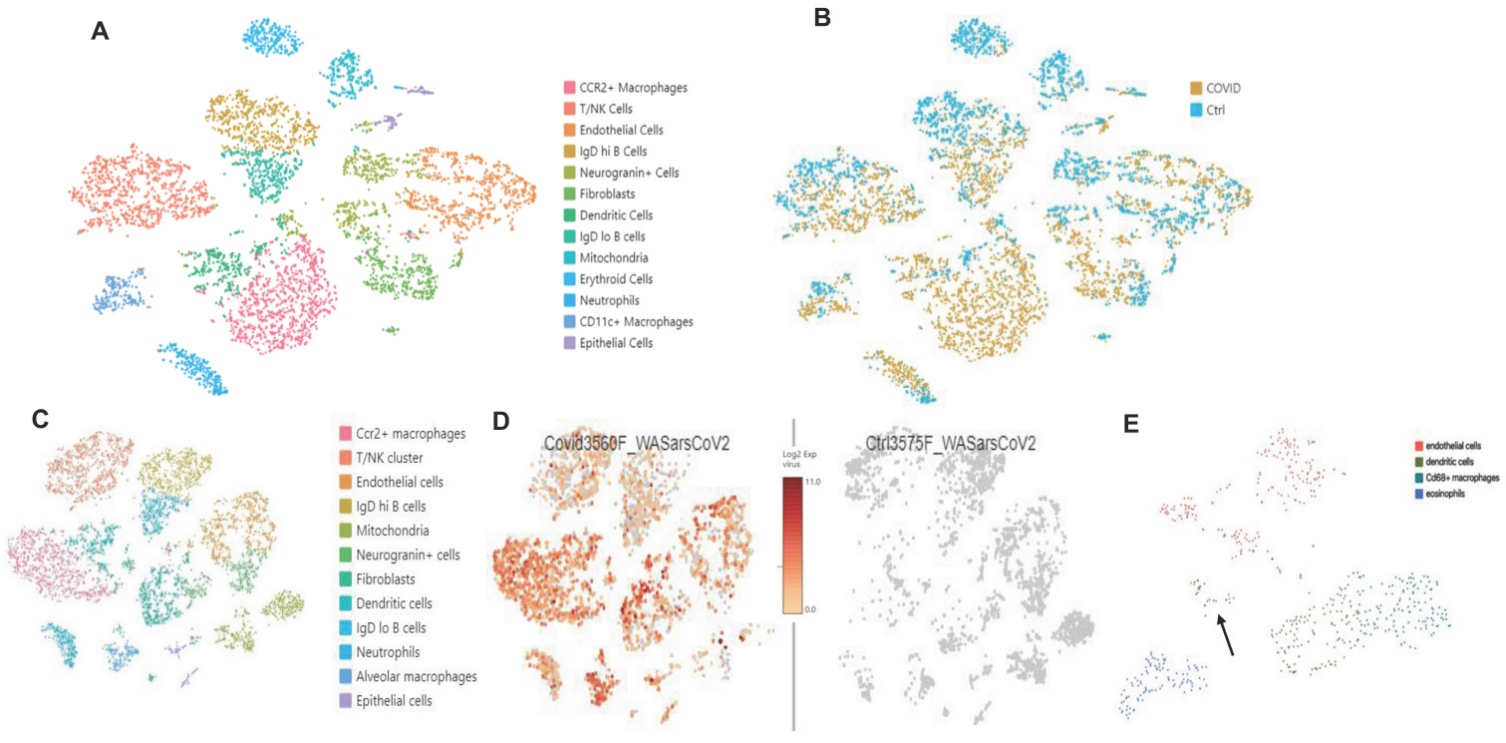
** scRNA-seq analysis in the *K18* model**. **(A. B)**: Increased CCR2+ Mϕ cluster, neutrophils, and fibroblast population. **(A)** TSNE plot and cluster analysis of lung cells day 4 post SARS-CoV-2 infection in WT and *K18* mice. **(B)** Separation of infected *K18* versus WT mice shows an increase in the CCR2+ Mϕ cluster, neutrophils, and fibroblast populations. **(C. D)**: Mapped viral RNA in each of the cell cluster.** (C)** Re-clustered TSNE plot of cells after dual mapping to the host and viral genome. (**D**) detection of mapped viral RNA in each of the cell clusters. (**E**) Using the re-clustering tool in Loupe Browser 5.0, we re-clustered the single cell data on a minimal virus expression log 2 intensity of 2, at least 1000 UMIs per cell, and a maximum of 6000 UMIs (based on distribution of the number of UMIs per cell) to avoid duplicates. The reclustering identified 4 main clusters with viral RNA: endothelial cells, dendritic cells, Cd68+ macrophages and eosinophils. Epithelial cells are indicated by the arrow.

Bulk RNAseq analyses of SARS-CoV-2 challenged *K18* vs WT mice showed up-regulation of 1078 genes (DESeq) and downregulation of 689 genes in *K18* mice compared to the infected littermate controls. Lung tissue mRNA array analysis at 3 and 6 PDI showed induction of inflammatory cytokines and chemokines including Cxcl10, Cxcl9, Cxcl13, IL-6, and CCL2 with prominent increases at 6 DPI (**Figure S9A-B**). Consistently, we only detected induction of IL-18, CxcL10 and CCL2 in sera collected from the infected *K18* mice compared to the infected WT mice at 6 but not at 4 DPI (**Figure S9C**). Pathway analysis showed induction of both type I and type II interferons, inflammatory response, and TNF-alpha signaling via NF-κB pathways, the IL-6/STAT3 pathway, and the K-ras pathway, as well as complement and coagulation at 4 and 6 DPI in SARS-CoV-2 infected *K18* mice compared to the infected WT mice (**Figure 6A-E, Figure S10A**). We performed over-enrichment analysis of the genes *Icam1, Vcam1* and *Cdh5*, which are respectively encoded for Icam1, Vcam1, and VE-cadherin proteins, all of which are the biomarker used for detecting EC dysfunction. The analyses revealed increased expression of Icam-1 and Vcam1 and reduced expression of Cdh5 in *K18* mice compared to the WT mice after challenge with SARS-CoV-2 (**Figure S10B**). These results further support the notion that SARS-CoV-2 infection mediates EC dysfunction. We previously observed induction of the interferon-dependent chemokines Cxcl9 and Cxcl10 in the Ad5-ACE2 model 34, as was also observed in the *K18* model. Single cell RNA-seq (scRNA-seq) analysis was performed at 4 DPI, which identified 12 distinct clusters of cells (**Figure 7A**).

scRNA-seq comparison between SARS-CoV-2 infected *K18* and WT mice showed an increase in the Cx3cr1/Ccr2+ macrophage (Mϕ) cluster (**Figure S11**), fibroblast cluster, and the neutrophil cluster, as well as IgDlo B cells and a trend towards increased type II pneumocytes (**Figure 7A-B**). We next analyzed data with a dual alignment to the mouse genome and the SARS-CoV-2 genome (WA1/2020 isolate, accession MN985325). TSNE analysis identified the same cell clusters in this algorithm. When mapped to the SARS-CoV-2 genome and analyzed by reclusterring tool in Loupe Browser 5.0, we found viral RNA in the following clusters: epithelial cells, endothelial cells, dendritic cells, Cd68+ macrophages and eosinophils (**Figure 7C-E**). Observation of viral RNA in ECs further supports our findings that the ECs can be infected by SARS-CoV-2. Our data also demonstrated greater expression of Cxcl9 and Cxcl10 in *Twist2*+ fibroblasts as well as in the EC cluster in SARS-CoV-2 infected *K18* mice compared to infected WT mice (**Figure S12**) indicative of an upregulated interferon pathway. Upregulation of the K-ras pathway was also observed (**Figure 6A-C**), as we detected the expression of several *serpin* genes in the scRNAseq data (**Figure S13A-C**). These analyses revealed that the infected *K18* expressed higher *Serpina3f* and* 3n* in the fibroblast and EC cluster (**Figure S13A-C**) and higher *Serpina3g* was expressed in these cell clusters in addition to the *Ccr2+* Mϕ cluster, the B cell cluster, and the T cell cluster (**Figure S13B**) compared to the infected WT mice. Given that there was evidence of interferon dependent chemokine expression in the EC cluster as well as evidence of EC activation (**Figures 3** and **5**), we analyzed expression of *Vcam1* and* Icam1* in the scRNA-seq data. Both molecules were expressed and up regulated in the EC and fibroblast clusters as well as the Mϕ/MC cluster in the infected *K18* mice (**Figure S13D-E**). These results indicate that Icam1 is also expressed in B cells, T cells, neutrophils and Cd11c+ Mϕ (**Figure S13E**). To further investigate whether specific pathways were regulated in distinct specific cell clusters by the presence of viral RNA, we analyzed viral positive versus negative cells in the endothelial and monocyte cluster. In this analysis, we found upregulation of genes in the lysosome/apoptosis and complement pathways in viral RNA positive cells (**Figure S14**). Together, these results shed lights on the importance of SARS-CoV-2-mediated immune activation for the endothelial dysfunction and injury.

Consistent with the bulk RNA-Seq data showing upregulation of the complement pathway, we observed that *Ctss*, *C1qa* and *C1qb* were highly expressed in the alveolar Mϕ cluster as well as the Cxc3r1/Ccr2+ Mϕ/MC cluster in SARS-CoV-2 infected *K18* mice compared to infected WT mice (**Figure S15A-D**). *Cfb* was also expressed in the same cells, as well as in the fibroblast cluster (**Figure S15**). Consistently, we also found increased C3 and a component of the membrane attack complex (MAC or C5b9) in the lung and ECs of SARS-CoV-2-infected *K18* mice and AGMs compared to the infected WT mice and naïve AGMs (**Figure S16**).

## Discussion

Most current COVID-19 animal models including hamsters, other human ACE2 transgenic mice and non-human primates at young ages, show only a mild to moderate COVID-19 phenotype. We recently reported a severe COVID-19 phenotype in aged AGMs 48. In contrast, SARS-CoV-2-infected *K18* mice at various ages are a newly established mouse model and have been successfully used for preclinical assessment of therapeutic and vaccine efficacy 49-51. In agreement with previous reports, we demonstrate that SARS-CoV-2 infected *K18* mice at two to three months of age exhibit many features of severe COVID-19 seen in patients associated with severe lung histological changes including severe interstitial pneumonia with progressive perivascular inflammation, edema, hemorrhage, and platelet activation and aggregation. Mice exhibited at least one or more of these pulmonary histopathologic alterations. Thus, we recommend incorporating the entirety of histopathologic changes for scoring or quantitative analysis when applying this severe model for evaluating therapeutic and vaccine efficacy at a histological level. Additionally, in a previous study, flow analysis of peripheral blood mononuclear cells (PBMCs) showed marked decrease in the lymphocytes, including CD4+, CD8+ T and B cells in this severe model 22. In the current study, by CBC count, we also observed lymphocytopenia in this model and further document that the infected mice develop eosinopenia as well. Both lymphocytopenia and eosinopenia are common findings in COVID-19 patients upon admission to the intensive care unit (ICU) 52, 53. Further, infection in this model also triggered mild damage to extrapulmonary organs including cerebral infarction, myocarditis, and hepatitis, which are consistent with previous findings 21, 22. Here, we also provide evidence that SARS-CoV-2 infection of the lung, not the brain, is the major cause of death in these mice. Previous studies have also suggested that the infection of the lungs in this severe COVID-19 model is a main cause of death. In contrast, two recent studies indicated that neuroinvasion and brain inflammation following SARS-CoV-2 infection is associated with fatality in *K18* mice 54, 55. Our results suggest that lung infection is the major cause of death in this severe COVID-19 model, reinforcing the notion that the *K18* mice infected with a moderate dose of SARS-CoV-2 represent an optimal model of severe COVID-19 for preclinical and mechanistic studies. Although COVID-19 is considered primarily as a respiratory disease, increasing clinical evidence also shows a large number of COVID-19 patients experience neurological symptoms such as anosmia and ageusia 56. Autopsies of deceased COVID-19 patients suggest that SARS-CoV-2 produces pathological changes in CNS by either direct infection of the brain or indirect cytokine storm 57, 58. The severe lung disease accompanied with CNS infection in *K18* mice is also consistent with clinical observations of severe cases of COVID-19, suggesting this model of a lower, sub-lethal dose of SARS-CoV-2 may be used for COVID-19-related CNS malfunction studies.

In this severe COVID-19 model, we identified previously recognized and un-recognized massive immune cell activation, infiltration of the lung and an array of cytokine and chemokine changes in the lung and circulation. We identified the overexpression of genes encoding for *Ccl2*, *Ccl3*, *Ccl4*, Ccl7, *Ccl17*, *Cxcl9*, *Cxcl10*, *Cxcl13* in the lung and increased serum levels of Ccl2, Cxcl10 and IL-18 related to the infiltration and activation of immune cells specifically for inflammatory Mϕ/MC. These results are comparable with data reported in autopsy samples 59, PBMCs 60, 61, and bronchoalveolar lavage 62 from severe COVID-19 patients. In addition, we observed upregulation of type I and type II interferons, inflammatory response, and TNF-alpha signaling via NF-κB and the IL-6/STAT3 pathways. These findings are consistent with the observation obtained from bulk RNA-seq analysis and RT-PCR analysis of the lungs of infected *K18* mice 22 and serum cytokine and chemokine arrays 20, 21, 63. Further, our scRNA-seq analysis showed a *Cx3cr1/Ccr2+* Mϕ cluster, a neutrophil cluster, an IgDlo B cluster, and a trend towards increased type II pneumocytes in infected *K18* mice. These data suggest that myeloid cell infiltration and activation contribute to the pathogenesis of severe COVID-19 similar to severe influenza pneumonia. scRNA-seq analysis also showed an increase in the fibroblast cluster, which has not been reported in COVID-19 patients or animal models before. It has recently been recognized that exuberant fibroblast activity compromises lung function via ADAMTS4 64. The increased activity of damage-responsive lung fibroblasts has been documented to drive lethal immunopathology during severe influenza virus infection 64. It remains to be determined if this pathway overlaps with COVID-19. Lack of the recognition of this finding in COVID-19 patients can be attributed to non-accessibility to their lungs in the early course of the disease and difficulty in conducting scRNA analysis of COVID-19 animal models under BSL3 restrictions. Whether the massive immune activation, including myeloid cell infiltration and activation, is linked to the increase in fibroblast cluster and how the increase in fibroblast cluster contributes to severe COVID-19 disease, remains unclear and requires further investigation.

We show that SARS-CoV-2 directly infects ECs in entire course of the COVID-19 disease in this severe murine and NHP COVID-19 models. Previous studies using these mice have mainly focused on SARS-CoV-2-induced lung histology changes and host immune responses rather than ECs 20-22, 65. Although early autopsy studies showed the presence of virus in ECs by EM 3, 8, concerns have been raised due to the difficulty in distinguishing virus particles from cellular structures in these studies 17, 66. In a COVID-19 patient autopsy cohort study, there was no evidence of viral RNA detectable in ECs 67. However, a recent study using in situ hybridization (ISH) to detect SARS-CoV-2 RNA found EC infection in 2 out of 32 cases 68. Other autopsy studies also showed the presence of virus in ECs of the stomach, ileum, liver 69 and heart 70. Since autopsies only reveal infection characteristics at late stages of COVID-19, during which infected cells are sporadically present 69, the infection of endothelium may be underestimated. A recent *in vitro* study suggests that SARS-CoV-2 can infect and induce a pro-inflammatory response in primary human ECs, even without productive viral infection 71. The strength of our study is the demonstration of SARS-CoV-2-infection of ECs using multiple complementary approaches, at both early and late stages of the disease and in a severe COVID-19 model. The functional consequence of the infection and the molecular mechanisms underlying the infection warrant further investigation. Further, we demonstrate that vast majority of endothelial cells infected by SARS-CoV-2 are alveolar capillary endothelial cells. Endothelial cells of *K-18* mice express very low-level of hACE2 as compared with lung epithelial cells such as club and alveolar type (AT2) cells (**Figure S17**), which are similar to human endothelial cells 1, 72. Whether hACE2 or mouse ACE2 in *K-18* mice facilitate the alveolar capillary endothelial cell infection remains unknown and requires further investigation. As lung epithelial cells shed virus, the local SARS-CoV-2 levels increase dramatically. Alveolar epithelial cells and endothelial cells of the alveolar capillaries are in direct contact with one another which, may facilitate infection of endothelial cells by SARS-CoV-2 viral particles released by lung epithelial cells. There is also possible that similar to other intracellular pathogens such as listeria monocytogenes 73, 74, SARS-CoV-2 virus may be directly transmitted from epithelial cells to alveolar capillary endothelial cells through cell-cell contacts. This intercellular pathogen trafficking may enable infection of cells that express low level receptor such as hACE-2 for infectious virus.

Using the lungs of two severe COVID-19 models (*K18* mice and AGMs 48) together with samples from autopsied patient, we demonstrate extensive pulmonary EC activation and dysfunction and increased vascular permeability, which explains the severe lung histopathology seen in these models including edema, hemorrhage and inflammatory and platelet activation. We used well-established cellular markers including increased ICAM and VCAM and decreased EV-cadherin, as markers of EC dysfunction. These biomarker changes in COVID-19 mouse models were further observed by heat map analysis of our bulk RNA-seq. Our findings showing lung EC dysfunction and damage are consistent with another study of SARS-CoV-2-infected rhesus macaques, which develop a mild COVID-19 disease 18. Moreover, we identify multiple pathways that may be associated with infection-mediated EC dysfunction and activation. First, we found that complement pathway activation may contribute to the EC dysfunction and activation in severe COVID-19. We observed increased complement C3 and MAC deposition in the EC layer of pulmonary vasculature. We also found increased transcript levels of C1qa, C1qb and Cfb in immune cells (mainly in Mϕ/MC), which are critical complement pathway components for complement activations. These results directly support the notion of massive acute and ongoing complement activation in the infected lung, which may contribute to EC dysfunction and activation. This prediction is further supported by previous studies by our group and others demonstrating that by products of complement activation contribute to pathogenesis of a variety of vascular diseases including atherosclerosis 75-77, ischemia and reperfusion injury 78, hyperacute graft rejection, vasculitis, hemolytic anemia 79-81, and the vascular complications of human diabetes 82. Second, we found evidence of extensive platelet and coagulation pathway activation in the lung. Previously, we reported the critical roles of platelet activation and coagulation pathway activation in fatal pulmonary arterial hypertension in a murine hemolysis model 27, 29. Together, these findings indicate that platelet activation and coagulation pathways could be direct consequences of EC infection and dysfunction and could also further exacerbate EC injury. Finally, we show the upregulation of the K-ras pathway in the fibroblast and EC clusters, a phenomenon previously unrecognized in COVID-19 patients and animal models. The effectors of K-ras signaling activated by SARS-CoV-2 infection all appear to mediate EC activation and permeability which play a critical role in cancer metastasis rather than cell proliferation, the other well-established effector of Kras signaling 83-85. The endothelial dysregulation of serpina3n has been found in atherosclerosis and aneurysm disease 86 and serpina3n attenuates rupture in a murine model of aortic aneurysm 87. Serpina3f, Serpina3g, and serpina3n are up regulated in blood brain barrier (BBB) in the mouse multiple sclerosis model 88. These findings indicate that SARS-CoV-2 infection directly or indirectly activates molecular pathways that may lead to increased vascular permeability and loss of EC barrier. Such a breach of EC permeability would have dramatic consequences for COVID-19 patients as the absence of the EC barrier virus may allow the virus to infect smooth muscle cells and cardiomyocytes which express ACE-2. The causative effect of the immune cell activation and the critical role of these cytokine and specific pathways on SARS-CoV-2 infection-mediated EC infection and dysfunction require further investigation.

In summary, we document SARS-CoV-2 infection-mediated massive immune activation, EC infection and dysfunction in a severe COVID-19 mouse. Our study sheds light on host viral interaction and highlights the pathogenic role of EC infection and dysfunction in the pathogenesis of severe COVID-19.

## Methods

### Mice and ethics statement

All animal experiments were approved by the Institutional Animal Care and Use Committees at Tulane University. *K18^+/-^*(034860) and *C57BL/6J* (000664) mice were purchased from the Jackson Laboratory and housed in the animal facility of Tulane University.

### SARS-CoV-2 strain and infection

We used SARS-CoV-2, Isolate USA-WA1/2020, NR-52281 deposited by the Centers for Disease Control and Prevention and obtained through BEI Resources, NIAID, NIH. We passaged the virus in VeroE6 cells in DMEM media with 2% FBS and sequenced the virus for verification as described previously 34. The harvested stock was determined to be 1.00E+6 TCID50/mL. Mice were intranasally infected by SARS-CoV-2(2 X 10^5^ TCID or 1 X 10^5^ TCID50) in ABSL3.

**Histological analysis and quantification of histopathologic lesions.** Sections of lung were processed routinely, stained with hematoxylin and eosin (H&E), and digitally scanned with a Zeiss Axio Scan.Z1. Inflammation and edema were quantified as previously described 34 and visually confirmed by a veterinary pathologist. Briefly, the stained lung sections were digitally scanned by a Zeiss Axio Scan.Z1 creating whole-slide images, and analyzed by a board certified pathologist with computer software (HALO v3.1, Indica Labs) using two algorithms (Multiplex IHC v2.3.4 and Spatial Analysis). Annotation regions were drawn around small arterioles, then all nucleated cells were counted using Multiplex IHC v2.3.4. Spatial analysis was used to quantify the number of nucleated cells within a 100 μm radius of the annotated vessels. Perivascular inflammation was reported as the density of nucleated cells within 100 μm radius of the tunica adventitia of small arterioles (nucleated cells/mm^2^).

### RNA isolation

Tissues were collected in 1mL Trizol reagent (15596026; Invitrogen), and extracted with RNeasy Mini Kit (Cat. No.74104; QIAGEN) following the manufacturer's protocol. The concentration of RNA was determined by NanoDrop 2000.

### Subgenomic N viral copy number detection

100ng total RNAs were mixed in Taqpath 1-Step Multiplex Master Mix (Cat. No.A15299; Thermo Fisher) and FAM-labeled primers (sgm-N-FOR: 5'-CGATCTCTTGTAGATCTGTTCTC-3', sgm-N-Probe: 5'-FAM-TAACCAGAATGGAGAACGCAGTGGG-TAMRA-3', sgm-N-reverse: 5'-GGTGAACCAAGACGCAGTAT-3'), following the manufacturer's instructions. Subgenomic N viral copy number was calculated by standard Cq values. The assay was performed under ABI QuantStudio 6 system.

### Tissue mRNA array

RT² Profiler™ PCR Array Mouse Cytokines & Chemokines (PAMM-150Z, Qiagen, Hilden, Germany) was used to determine the expression of 84 cytokines and chemokines as previously decribed 89. The assay was performed under ABI QuantStudio 6 system.

### Bulk RNA sequencing

Lung tissue RNA was used to perform RNA sequencing. RNA concentration was determined with a Qubit 3.0 Fluorometer (ThermoFisher Scientific). RNA integrity number and fragment sizes (DV200 metrics) were obtained with either the Agilent 2100 Bioanalyzer or the Agilent 4150TapeStation. Illumina TruSeq Stranded mRNA sample prep kit was used for library preparation. The cDNA libraries were pooled at a final concentration of 1.8 pM for cluster generation and sequenced using NextSeq 500/550 High Output Kit (150 Cycles) on an Illumina NextSeq 550 using a minimum of 4 ng of RNA according to SMART-Seq Stranded Kit User Manual (Takara Bio USA, Inc). Raw reads were processed and mapped, then gene expression and nucleotide variation were evaluated as previously described 90, 91. Raw read counts were normalized across all samples and then used for differential expression analysis using DESeq, EdgeR and Cuffdiff (Slug Genomics, UV Santa Cruz). The EdgeR output was used to generate volcano plots in R. Data was deposited in the Sequence Read Archive BioProject number: GSE175996.

**Over-enrichment analysis.** Enrichment analysis was performed with the R package 'ClusterProfiler' at default parameters. Enrichment scores were calculated against the following four pathway databases containing a priori-defined gene sets: Gene Ontology (GO) database, Hallmark (H) and Curated (C2) gene sets of the Molecular Signature database (MsigDB), and WikiPathways. Gene sets significantly enriched in the datasets (p < 0.05) were subsequently curated for those relevant to EC biological function. Enrichment plot was generated with the R software package ggplot2 and heatmaps with the ComplexHeatmap package.

**Single cell RNA-seq analysis.** Lungs were isolated and minced with forceps and scissors. They were further enzymatically digested with 2 mg/mL collagenase (Sigma) and 80 U/mL DNase I (Sigma) in 2 mL serum-free medium for 60 min at 37°C. Digested tissues were passed through a sterile 70 mm filter (Fisher Scientific) and 40 mm filter (Miltenyi Biotec) to generate a single cell suspension. After washing, cell suspensions were incubated with 2 ml of ACK Lysing Buffer (Cat #A1049201, Gibco) for 2 min and washed twice. 1x10^6^ cells per condition were collected as whole lung single cell population. Cell numbers and viability were validated by Countess II (Thermo Fisher) prior to preparation of single cell RNAseq library.

**10x single-cell RNA-seq assay**. 5000 live cells per sample were targeted by using 10x single-cell RNA-seq technology provided by 10x Genomics (10X Genomics Inc, CA). Briefly, viable single cell suspensions were partitioned into nanoliter-scale Gel Beads-In-EMulsion (GEMs). Full-length barcoded cDNAs were then generated and amplified by PCR to obtain sufficient mass for library construction. Following enzymatic fragmentation, end-repair, A-tailing, and adaptor ligation, single cell 3' libraries comprising standard Illumina P5 and P7 paired-end constructs were generated. Library quality controls were performed using Agilent High Sensitivity DNA kit with Agilent 2100 Bioanalyzer and quantified by Qubit 2.0 fluorometer. Pooled libraries at a final concentration of 650 pM were sequenced with paired end dual index configuration by Illumina NextSeq 2000. Cell Ranger version 4.0.0 (10x Genomics) was used to process raw sequencing data and Loupe Cell Browser (10x Genomics) to obtain differentially expressed genes between specified cell clusters. In addition, Seurat suite version 2.2.1 90, 91 was used for further quality control and downstream analysis. Filtering was performed to remove multiplets and broken cells. Variable genes were determined by iterative selection based on the dispersion vs. average expression of the gene.

### Immunohistochemistry assay

5 um sections of paraffin-embedded lung were baked overnight at 60 ºC, then dewaxed and rehydrated with xylene, graded ethanol and dd water. Slides were retrieved using a microwave oven heating method in Tris-EDTA buffer (ab93684, Abcam) for 20 min and cooled to room temperature. Sections were incubated with endogenous blocking solution (SP-6000-100, vector lab) for 10 min and 2.5% normal horse serum for 20 min at room temperature. Primary antibodies were incubated overnight at 4 ºC, then the slides were incubated with HRP horse anti-Rabbit IgG reagent for 30 min at room temperature. Immunoreactivity was detected using DAB system. Slides were digitally scanned by Zeiss Axio Scan. Z1.

### RNA-scope

Z-fix-embedded lung sections were stained according to RNAscope® 2.5 HD Duplex detection kit guide. Z-fix-embedded lung sections were deparaffinized in fresh xylene and fresh 100% ethanol and then air dried. Target retrieval was performed (RNAscope® Target Retrieval Reagents, ACD Cat# 322000) after hydrogen peroxide treatment and was followed by protease treatment (RNAscope® H2O2 & Protease Plus, ACD Cat# 322330). Probes for RNAs of platelet endothelial cell adhesion molecule (RNAscope® Probe *Mm-pcam1*, ACD Cat# 316721) and *spike* protein (RNAscope® Probe *V-nCoV2019-S-C2*, ACD Cat# 848561-C2) were mixed and incubated on slides for *in situ* hybridization. Finally, slides were stained with 50% hematoxylin (Hematoxiylin Solution, Gill No.1, Sigma-Aldrich, Cat# GHS132) and mounted with VectaMount Permanent Mounting Media (ACD, Cat# 321584). Slides were digitally scanned by Zeiss Axio Scan.Z1.

### Immunostaining

Immunostaining was performed as described previously 34. Zinc formalin fixed (Z-fixed), paraffin embedded lung was sectioned, deparaffinized, and subjected to heat induced epitope retrieval using both high-pH (Vector Labs H-3301), and low-pH (Vector Labs H-3300) solutions. Sections were blocked with 10% BSA or 1% donkey serum for 40 min, incubated with the primary antibodies for overnight at 4 ^0^C and secondary antibodies for 40 min at room temperature (**Table S2**). Slides were digitally scanned by Zeiss Axio scan. Z1.

### Scanning EM

Lung tissues were inflated and fixed with 2.5% glutaraldehyde buffer, washed 3 times with fresh cacodylate buffer (10 min each time), and postfixed by 1% OsO4 for 1 h at room temperature. Specimens were dehydrated in graded ethanol (30, 50, 70, 90% and 100%). Images were taken with Hitachi 4800 SEM.

### Transmission EM

1mm^3^ lung cubes were fixed with 2.5% glutaraldehyde buffer followed by 1% OsO4. After dehydration, 90 nm thin sections were stained with UranyLess and lead citrate. Images were taken with JEM-1400.

### Statistics

Data are shown as mean ± SEM. To compare values obtained from multiple groups over time, two-way analysis of variance (ANOVA) was used, followed by Bonferroni post hoc test. To compare values obtained from two groups, the unpaired Student's *t*-test was performed. Statistical significance was taken at the P < 0.05 level.

## Supplementary Material

Supplementary figures and tables.

## References

[1] Datta PK, Liu F, Fischer T, Rappaport J, Qin X (2020). SARS-CoV-2 pandemic and research gaps: Understanding SARS-CoV-2 interaction with the ACE2 receptor and implications for therapy. Theranostics.

[2] O'Sullivan JM, Gonagle DM, Ward SE, Preston RJS, O'Donnell JS (2020). Endothelial cells orchestrate COVID-19 coagulopathy. Lancet Haematol.

[3] Ackermann M, Verleden SE, Kuehnel M, Haverich A, Welte T, Laenger F (2020). Pulmonary Vascular Endothelialitis, Thrombosis, and Angiogenesis in Covid-19. N Engl J Med.

[4] Perico L, Benigni A, Casiraghi F, Ng LFP, Renia L, Remuzzi G (2021). Immunity, endothelial injury and complement-induced coagulopathy in COVID-19. Nat Rev Nephrol.

[5] Evans PC, Rainger GE, Mason JC, Guzik TJ, Osto E, Stamataki Z (2020). Endothelial dysfunction in COVID-19: a position paper of the ESC Working Group for Atherosclerosis and Vascular Biology, and the ESC Council of Basic Cardiovascular Science. Cardiovasc Res.

[6] Mondal R, Lahiri D, Deb S, Bandyopadhyay D, Shome G, Sarkar S (2020). COVID-19: Are we dealing with a multisystem vasculopathy in disguise of a viral infection?. J Thromb Thrombolysis.

[7] Gu SX, Tyagi T, Jain K, Gu VW, Lee SH, Hwa JM (2020). Thrombocytopathy and endotheliopathy: crucial contributors to COVID-19 thromboinflammation. Nat Rev Cardiol.

[8] Varga Z, Flammer AJ, Steiger P, Haberecker M, Andermatt R, Zinkernagel A (2020). Electron microscopy of SARS-CoV-2: a challenging task - Authors' reply. Lancet.

[9] Goshua G, Pine AB, Meizlish ML, Chang CH, Zhang H, Bahel P (2020). Endotheliopathy in COVID-19-associated coagulopathy: evidence from a single-centre, cross-sectional study. Lancet Haematol.

[10] Fox SE, Akmatbekov A, Harbert JL, Li G, Quincy Brown J, Vander Heide RS (2020). Pulmonary and cardiac pathology in African American patients with COVID-19: an autopsy series from New Orleans. Lancet Respir Med.

[11] Dolhnikoff M, Ferreira Ferranti J, de Almeida Monteiro RA, Duarte-Neto AN, Soares Gomes-Gouvea M, Viu Degaspare N (2020). SARS-CoV-2 in cardiac tissue of a child with COVID-19-related multisystem inflammatory syndrome. Lancet Child Adolesc Health.

[12] Mangalmurti NS, Reilly JP, Cines DB, Hunter CA, Meyer NJ, Vaughan AE (2020). COVID-ARDS Clarified: A Vascular Endotype?. Am J Respir Crit Care Med.

[13] Libby P, Luscher T (2020). COVID-19 is, in the end, an endothelial disease. Eur Heart J.

[14] Kaur S, Tripathi DM, Yadav A (2020). The Enigma of Endothelium in COVID-19. Front Physiol.

[15] Goldsmith CS, Miller SE (2020). Caution in Identifying Coronaviruses by Electron Microscopy. J Am Soc Nephrol.

[16] Roufosse C, Curtis E, Moran L, Hollinshead M, Cook T, Hanley B (2020). Electron microscopic investigations in COVID-19: not all crowns are coronas. Kidney Int.

[17] Dittmayer C, Meinhardt J, Radbruch H, Radke J, Heppner BI, Heppner FL (2020). Why misinterpretation of electron micrographs in SARS-CoV-2-infected tissue goes viral. Lancet.

[18] Aid M, Busman-Sahay K, Vidal SJ, Maliga Z, Bondoc S, Starke C (2020). Vascular Disease and Thrombosis in SARS-CoV-2-Infected Rhesus Macaques. Cell.

[19] McCray PB Jr, Pewe L, Wohlford-Lenane C, Hickey M, Manzel L, Shi L (2007). Lethal infection of K18-hACE2 mice infected with severe acute respiratory syndrome coronavirus. J Virol.

[20] Golden JW, Cline CR, Zeng X, Garrison AR, Carey BD, Mucker EM (2020). Human angiotensin-converting enzyme 2 transgenic mice infected with SARS-CoV-2 develop severe and fatal respiratory disease. JCI Insight.

[21] Oladunni FS, Park JG, Pino PA, Gonzalez O, Akhter A, Allue-Guardia A (2020). Lethality of SARS-CoV-2 infection in K18 human angiotensin-converting enzyme 2 transgenic mice. Nat Commun.

[22] Winkler ES, Bailey AL, Kafai NM, Nair S, McCune BT, Yu J (2020). SARS-CoV-2 infection of human ACE2-transgenic mice causes severe lung inflammation and impaired function. Nat Immunol.

[23] Zheng J, Wong LR, Li K, Verma AK, Ortiz M, Wohlford-Lenane C (2020). COVID-19 treatments and pathogenesis including anosmia in K18-hACE2 mice. Nature.

[24] Rathnasinghe R, Strohmeier S, Amanat F, Gillespie VL, Krammer F, Garcia-Sastre A (2020). Comparison of Transgenic and Adenovirus hACE2 Mouse Models for SARS-CoV-2 Infection. Emerg Microbes Infect.

[25] Bradley BT, Maioli H, Johnston R, Chaudhry I, Fink SL, Xu H (2020). Histopathology and ultrastructural findings of fatal COVID-19 infections in Washington State: a case series. Lancet.

[26] Borczuk AC, Salvatore SP, Seshan SV, Patel SS, Bussel JB, Mostyka M (2020). COVID-19 pulmonary pathology: a multi-institutional autopsy cohort from Italy and New York City. Mod Pathol.

[27] Hu W, Ferris SP, Tweten RK, Wu G, Radaeva S, Gao B (2008). Rapid conditional targeted ablation of cells expressing human CD59 in transgenic mice by intermedilysin. Nat Med.

[28] Blann AD, Nadar SK, Lip GY (2003). The adhesion molecule P-selectin and cardiovascular disease. Eur Heart J.

[29] Hu W, Jin R, Zhang J, You T, Peng Z, Ge X (2010). The critical roles of platelet activation and reduced NO bioavailability in fatal pulmonary arterial hypertension in a murine hemolysis model. Blood.

[30] Huang G, Kovalic AJ, Graber CJ (2020). Prognostic Value of Leukocytosis and Lymphopenia for Coronavirus Disease Severity. Emerg Infect Dis.

[31] Liu J, Li H, Luo M, Liu J, Wu L, Lin X (2020). Lymphopenia predicted illness severity and recovery in patients with COVID-19: A single-center, retrospective study. PLoS One.

[32] Klein R, Nagy O, Tothova C, Chovanova F (2020). Clinical and Diagnostic Significance of Lactate Dehydrogenase and Its Isoenzymes in Animals. Vet Med Int.

[33] Wolfel R, Corman VM, Guggemos W, Seilmaier M, Zange S, Muller MA (2020). Virological assessment of hospitalized patients with COVID-2019. Nature.

[34] Han K, Blair RV, Iwanaga N, Liu F, Russell-Lodrigue KE, Qin Z (2021). Lung Expression of Human Angiotensin-Converting Enzyme 2 Sensitizes the Mouse to SARS-CoV-2 Infection. Am J Respir Cell Mol Biol.

[35] Monchatre-Leroy E, Lesellier S, Wasniewski M, Picard-Meyer E, Richomme C, Boué F (2021). Hamster and ferret experimental infection with intranasal low dose of a single strain of SARS-CoV-2. J Gen Virol.

[36] Munster VJ, Feldmann F, Williamson BN, van Doremalen N, Perez-Perez L, Schulz J (2020). Respiratory disease in rhesus macaques inoculated with SARS-CoV-2. Nature.

[37] Hassan AO, Case JB, Winkler ES, Thackray LB, Kafai NM, Bailey AL (2020). A SARS-CoV-2 Infection Model in Mice Demonstrates Protection by Neutralizing Antibodies. Cell.

[38] Rosenke K, Meade-White K, Letko M, Clancy C, Hansen F, Liu Y (2020). Defining the Syrian hamster as a highly susceptible preclinical model for SARS-CoV-2 infection. Emerg Microbes Infect.

[39] Varga Z, Flammer AJ, Steiger P, Haberecker M, Andermatt R, Zinkernagel AS (2020). Endothelial cell infection and endotheliitis in COVID-19. Lancet.

[40] Liao JK (2013). Linking endothelial dysfunction with endothelial cell activation. J Clin Invest.

[41] Hebda JK, Leclair HM, Azzi S, Roussel C, Scott MG, Bidere N (2013). The C-terminus region of beta-arrestin1 modulates VE-cadherin expression and endothelial cell permeability. Cell Commun Signal.

[42] Gavard J, Gutkind JS (2006). VEGF controls endothelial-cell permeability by promoting the beta-arrestin-dependent endocytosis of VE-cadherin. Nat Cell Biol.

[43] Hoffmann M, Kleine-Weber H, Schroeder S, Kruger N, Herrler T, Erichsen S (2020). SARS-CoV-2 Cell Entry Depends on ACE2 and TMPRSS2 and Is Blocked by a Clinically Proven Protease Inhibitor. Cell.

[44] Barbosa LC, Gonçalves TL, de Araujo LP, Rosario LVO, Ferrer VP (2021). Endothelial cells and SARS-CoV-2: An intimate relationship. Vascul Pharmacol.

[45] Lima MA, Skidmore M, Khanim F, Richardson A (2021). Development of a nano-luciferase based assay to measure the binding of SARS-CoV-2 spike receptor binding domain to ACE-2. Biochem Biophys Res Commun.

[46] Mycroft-West CJ, Su D, Pagani I, Rudd TR, Elli S, Gandhi NS (2020). Heparin Inhibits Cellular Invasion by SARS-CoV-2: Structural Dependence of the Interaction of the Spike S1 Receptor-Binding Domain with Heparin. Thromb Haemost.

[47] Blair RV, Vaccari M, Doyle-Meyers LA, Roy CJ, Russell-Lodrigue K, Fahlberg M (2021). Acute Respiratory Distress in Aged, SARS-CoV-2-Infected African Green Monkeys but Not Rhesus Macaques. Am J Pathol.

[48] Blair RV, Vaccari M, Doyle-Meyers LA, Roy CJ, Russell-Lodrigue K, Fahlberg M (2021). Acute Respiratory Distress in Aged, SARS-CoV-2-Infected African Green Monkeys but Not Rhesus Macaques. Am J Pathol.

[49] Rosenfeld R, Noy-Porat T, Mechaly A, Makdasi E, Levy Y, Alcalay R (2021). Post-exposure protection of SARS-CoV-2 lethal infected K18-hACE2 transgenic mice by neutralizing human monoclonal antibody. Nat Commun.

[50] Johnson BA, Xie X, Bailey AL, Kalveram B, Lokugamage KG, Muruato A (2021). Loss of furin cleavage site attenuates SARS-CoV-2 pathogenesis. Nature.

[51] Joaquín Cáceres C, Cardenas-Garcia S, Carnaccini S, Seibert B, Rajao DS, Wang J (2021). Efficacy of GC-376 against SARS-CoV-2 virus infection in the K18 hACE2 transgenic mouse model. bioRxiv. 2021 Jan 27.

[52] Guan WJ, Ni ZY, Hu Y, Liang WH, Ou CQ, He JX (2020). Clinical Characteristics of Coronavirus Disease 2019 in China. N Engl J Med.

[53] Lindsley AW, Schwartz JT, Rothenberg ME (2020). Eosinophil responses during COVID-19 infections and coronavirus vaccination. J Allergy Clin Immunol.

[54] Carossino M, Montanaro P, O'Connell A, Kenney D, Gertje H, Grosz KA (2021). Fatal neuroinvasion of SARS-CoV-2 in K18-hACE2 mice is partially dependent on hACE2 expression. bioRxiv.

[55] Kumari P, Rothan HA, Natekar JP, Stone S, Pathak H, Strate PG (2021). Neuroinvasion and Encephalitis Following Intranasal Inoculation of SARS-CoV-2 in K18-hACE2 Mice. Viruses.

[56] Conde Cardona G, Quintana Pajaro LD, Quintero Marzola ID, Ramos Villegas Y, Moscote Salazar LR (2020). Neurotropism of SARS-CoV 2: Mechanisms and manifestations. J Neurol Sci.

[57] Meinhardt J, Radke J, Dittmayer C, Franz J, Thomas C, Mothes R (2021). Olfactory transmucosal SARS-CoV-2 invasion as a port of central nervous system entry in individuals with COVID-19. Nat Neurosci.

[58] Song E, Zhang C, Israelow B, Lu-Culligan A, Prado AV, Skriabine S (2021). Neuroinvasion of SARS-CoV-2 in human and mouse brain. J Exp Med.

[59] Desai N, Neyaz A, Szabolcs A, Shih AR, Chen JH, Thapar V (2020). Temporal and spatial heterogeneity of host response to SARS-CoV-2 pulmonary infection. Nature Communications.

[60] Silvin A, Chapuis N, Dunsmore G, Goubet AG, Dubuisson A, Derosa L (2020). Elevated Calprotectin and Abnormal Myeloid Cell Subsets Discriminate Severe from Mild COVID-19. Cell.

[61] Zhu L, Yang P, Zhao Y, Zhuang Z, Wang Z, Song R (2020). Single-Cell Sequencing of Peripheral Mononuclear Cells Reveals Distinct Immune Response Landscapes of COVID-19 and Influenza Patients. Immunity.

[62] Zhang F, Mears JR, Shakib L, Beynor JI, Shanaj S, Korsunsky I (2021). IFN-γ and TNF-α drive a CXCL10+ CCL2+ macrophage phenotype expanded in severe COVID-19 lungs and inflammatory diseases with tissue inflammation. Genome Med.

[63] Yinda CK, Port JR, Bushmaker T, Offei Owusu I, Purushotham JN, Avanzato VA (2021). K18-hACE2 mice develop respiratory disease resembling severe COVID-19. PLoS Pathog.

[64] Boyd DF, Allen EK, Randolph AG, Guo X-zJ, Weng Y, Sanders CJ (2020). Exuberant fibroblast activity compromises lung function via ADAMTS4. Nature.

[65] Yinda CK, Port JR, Bushmaker T, Offei Owusu I, Purushotham JN, Avanzato VA (2021). K18-hACE2 mice develop respiratory disease resembling severe COVID-19. PLoS Pathog.

[66] Goldsmith CS, Miller SE, Martines RB, Bullock HA, Zaki SR (2020). Electron microscopy of SARS-CoV-2: a challenging task. Lancet.

[67] Szekely L, Bozoky B, Bendek M, Ostad M, Lavignasse P, Haag L (2021). Pulmonary stromal expansion and intra-alveolar coagulation are primary causes of COVID-19 death. Heliyon.

[68] Bhatnagar J, Gary J, Reagan-Steiner S, Estetter LB, Tong S, Tao Y (2021). Evidence of SARS-CoV-2 Replication and Tropism in the Lungs, Airways and Vascular Endothelium of Patients with Fatal COVID-19: An Autopsy Case-Series. J Infect Dis.

[69] Schurink B, Roos E, Radonic T, Barbe E, Bouman CSC, de Boer HH (2020). Viral presence and immunopathology in patients with lethal COVID-19: a prospective autopsy cohort study. Lancet Microbe.

[70] Fox SE, Li G, Akmatbekov A, Harbert JL, Lameira FS, Brown JQ (2020). Unexpected Features of Cardiac Pathology in COVID-19 Infection. Circulation.

[71] Schimmel L, Chew KY, Stocks C, Yordanov T, Essebier T, Kulasinghe A (2021). Endothelial cells elicit a pro-inflammatory response to SARS-COV-2 without productive viral infection. bioRxiv. 2021.

[72] Zhao Y, Zhao Z, Wang Y, Zhou Y, Ma Y, Zuo W (2020). Single-Cell RNA Expression Profiling of ACE2, the Receptor of SARS-CoV-2. Am J Respir Crit Care Med.

[73] Bahnan W, Boucher JC, Gayle P, Shrestha N, Rosen M, Aktas B (2018). The eIF2α Kinase Heme-Regulated Inhibitor Protects the Host from Infection by Regulating Intracellular Pathogen Trafficking. Infection and Immunity.

[74] Cifuentes-Munoz N, Dutch RE, Cattaneo R (2018). Direct cell-to-cell transmission of respiratory viruses: The fast lanes. PLoS Pathog.

[75] Wu G, Chen T, Shahsafaei A, Hu W, Bronson RT, Shi GP (2010). Complement regulator CD59 protects against angiotensin II-induced abdominal aortic aneurysms in mice. Circulation.

[76] Wu G, Hu W, Shahsafaei A, Song W, Dobarro M, Sukhova GK (2009). Complement regulator CD59 protects against atherosclerosis by restricting the formation of complement membrane attack complex. Circ Res.

[77] Liu F, Sahoo R, Ge X, Wu L, Ghosh P, Qin X (2017). Deficiency of the complement regulatory protein CD59 accelerates the development of diabetes-induced atherosclerosis in mice. J Diabetes Complications.

[78] Zhang J, Hu W, Xing W, You T, Xu J, Qin X (2011). The protective role of CD59 and pathogenic role of complement in hepatic ischemia and reperfusion injury. Am J Pathol.

[79] Atkinson JP, Goodship TH (2007). Complement factor H and the hemolytic uremic syndrome. J Exp Med.

[80] Qin X, Hu W, Song W, Blair P, Wu G, Hu X (2009). Balancing role of nitric oxide in complement-mediated activation of platelets from mCd59a and mCd59b double-knockout mice. Am J Hematol.

[81] Qin X, Krumrei N, Grubissich L, Dobarro M, Aktas H, Perez G (2003). Deficiency of the mouse complement regulatory protein mCd59b results in spontaneous hemolytic anemia with platelet activation and progressive male infertility. Immunity.

[82] Acosta J, Qin X, Halperin J (2004). Complement and complement regulatory proteins as potential molecular targets for vascular diseases. Curr Pharm Des.

[83] Salazar-Olivo LA, Mejia-Elizondo R, Alonso-Castro AJ, Ponce-Noyola P, Maldonado-Lagunas V, Melendez-Zajgla J (2014). SerpinA3g participates in the antiadipogenesis and insulin-resistance induced by tumor necrosis factor-α in 3T3-F442A cells. Cytokine.

[84] Ni H, Xu S, Chen H, Dai Q (2020). Nicotine Modulates CTSS (Cathepsin S) Synthesis and Secretion Through Regulating the Autophagy-Lysosomal Machinery in Atherosclerosis. Arterioscler Thromb Vasc Biol.

[85] Hu X, Zhang P, Xu Z, Chen H, Xie X (2013). GPNMB enhances bone regeneration by promoting angiogenesis and osteogenesis: potential role for tissue engineering bone. J Cell Biochem.

[86] Wågsäter D, Johansson D, Fontaine V, Vorkapic E, Bäcklund A, Razuvaev A (2012). Serine protease inhibitor A3 in atherosclerosis and aneurysm disease. Int J Mol Med.

[87] Ang LS, Boivin WA, Williams SJ, Zhao H, Abraham T, Carmine-Simmen K (2011). Serpina3n attenuates granzyme B-mediated decorin cleavage and rupture in a murine model of aortic aneurysm. Cell Death & Disease.

[88] Munji RN, Soung AL, Weiner GA, Sohet F, Semple BD, Trivedi A (2019). Profiling the mouse brain endothelial transcriptome in health and disease models reveals a core blood-brain barrier dysfunction module. Nat Neurosci.

[89] Dai S, Liu F, Qin Z, Zhang J, Chen J, Ding WX (2020). Kupffer cells promote T-cell hepatitis by producing CXCL10 and limiting liver sinusoidal endothelial cell permeability. Theranostics.

[90] Butler A, Hoffman P, Smibert P, Papalexi E, Satija R (2018). Integrating single-cell transcriptomic data across different conditions, technologies, and species. Nat Biotechnol.

[91] Macosko EZ, Basu A, Satija R, Nemesh J, Shekhar K, Goldman M (2015). Highly Parallel Genome-wide Expression Profiling of Individual Cells Using Nanoliter Droplets. Cell.

