# Supplementary material for: Endothelial cell infection and dysfunction, immune activation in severe COVID-19
Source: Theranostics. 2021 Jul 6;11(16):8076–91. doi: 10.7150/thno.61810 (PMC8315069; doi:10.7150/thno.61810)
Supplement: Supplementary file 1 — Supplementary figures and tables. [file thnov11p8076s1.pdf]

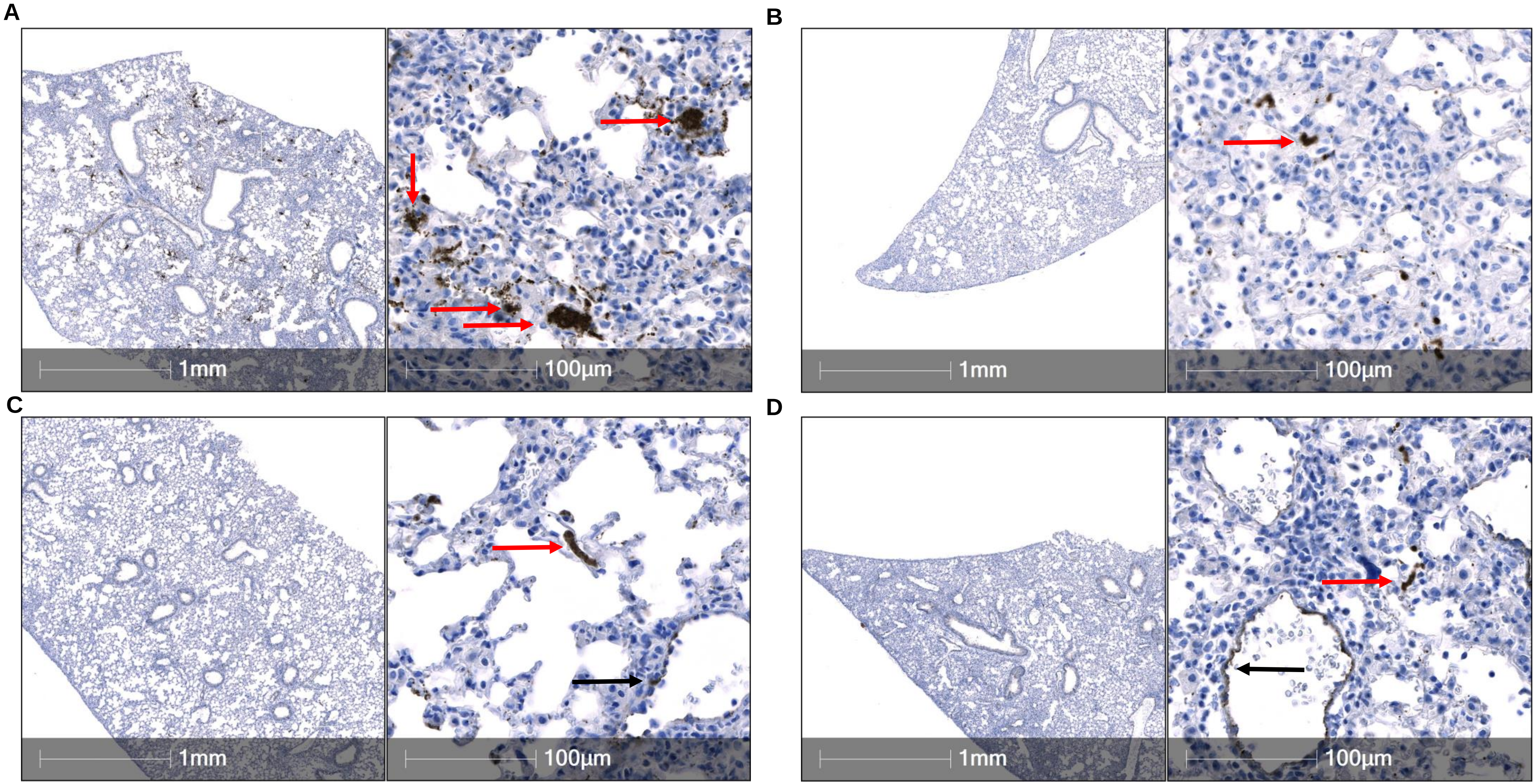

Figure S1. Thrombosis and activated platelets adhered to endothelial cells were detected by P-selectin in the K18-hACE2$^{+/-}$ lung at 6 days post infection (DPI). A. The mouse had a severe to moderate thrombosis in the lung**. B-D.** Three mice had mild thrombosis in the lungs. Red and black arrows point respectively the thromboses and activated platelets adhered to endothelial cells in some areas of the lung**.**

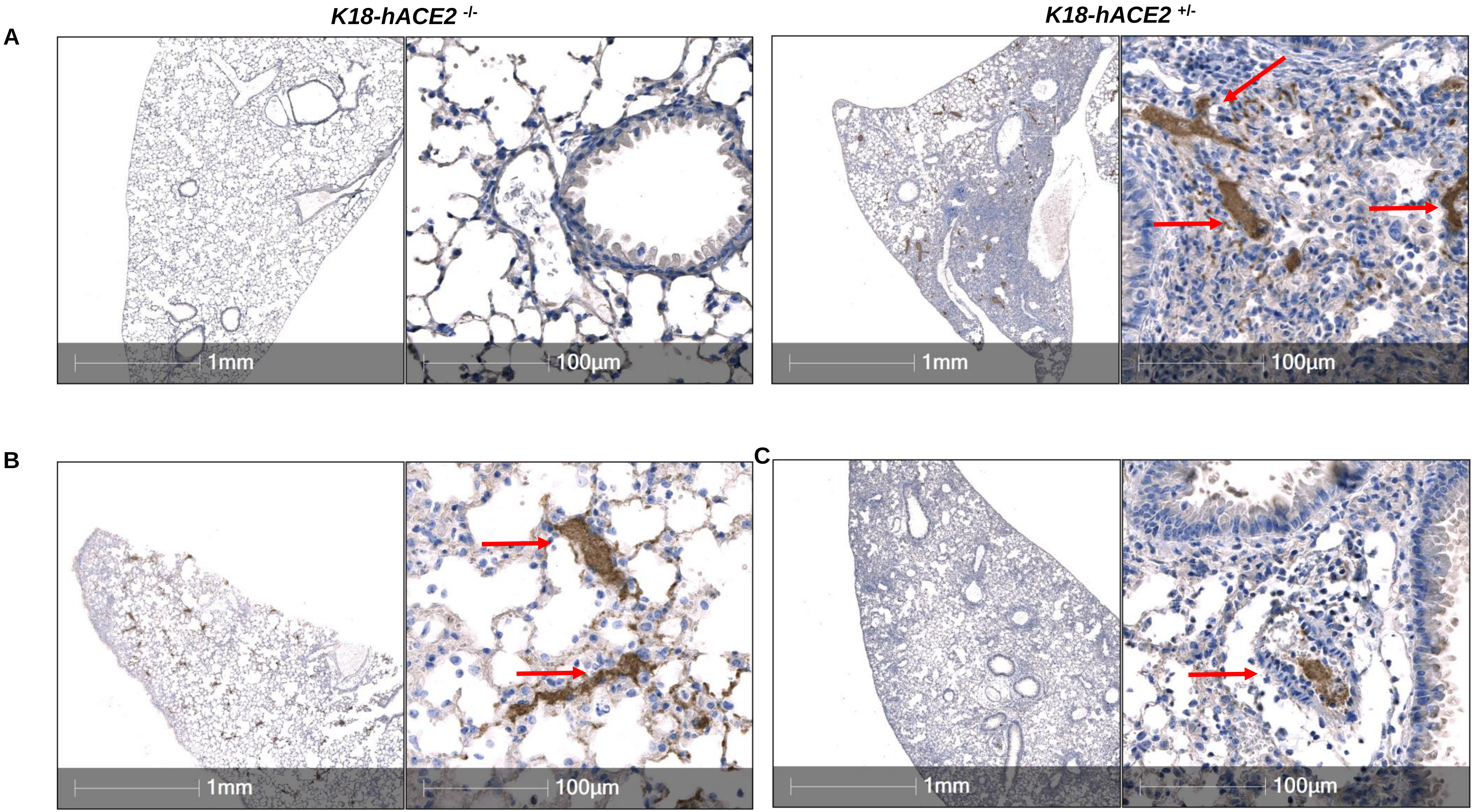

Figure S2. Thrombosis and activated platelets adhered to endothelial cells were detected by CD41 in the K18-hACE2$^{+/-}$ lung at 6 days post infection (DPI). A. The mouse had a severe to moderate thrombosis in the lung. **B-C.** Two mice had moderate to mild thrombosis in the lungs. Red arrows point the thromboses and activated platelets adhered to endothelial cells in some areas of the lung.

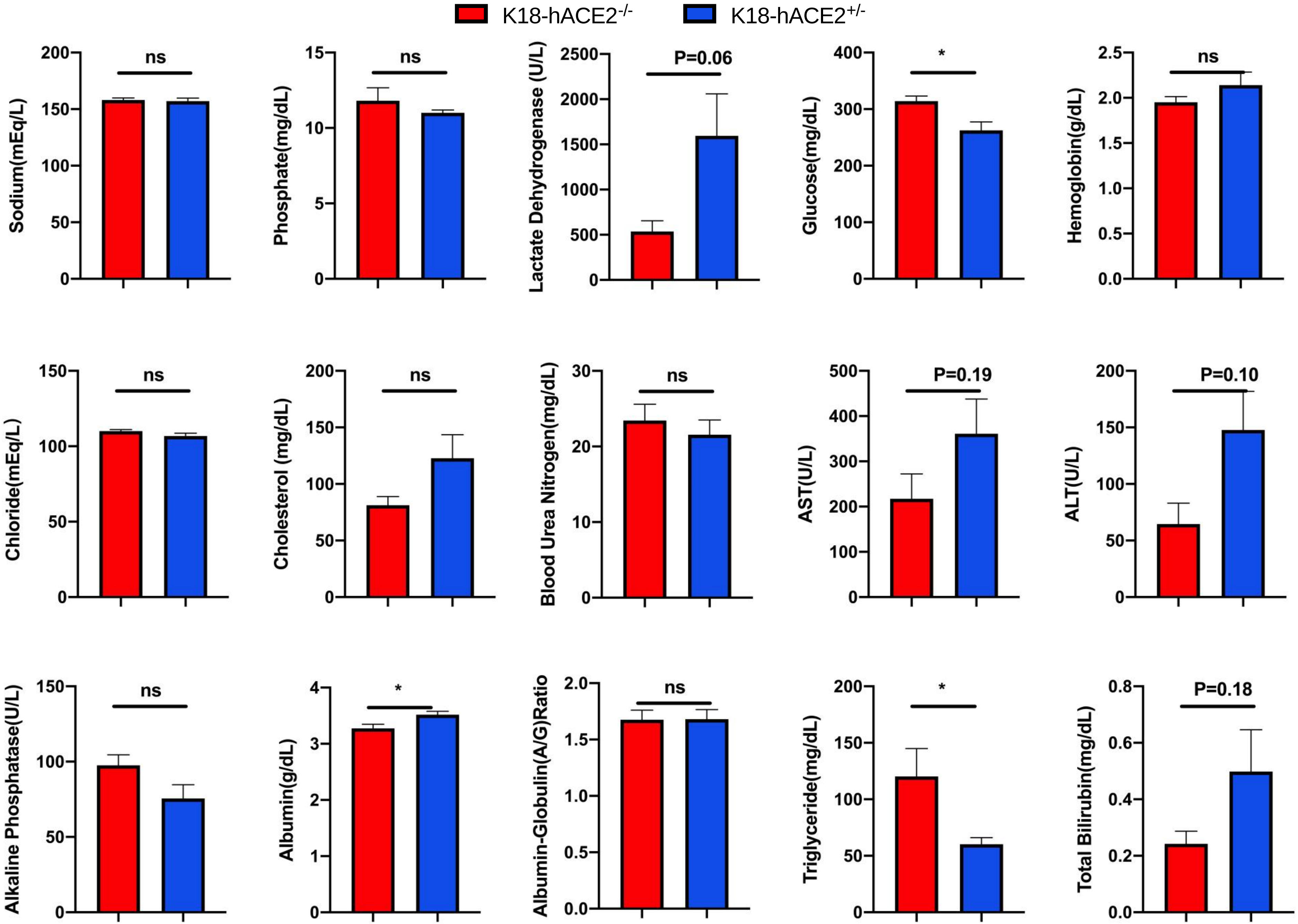

Figure S3. Serum chemistry analysis at 5 dpi . Analyzed by unpaired student t-test (N= 3 for *K18-hACE2*$^{-/-}$,N=6 for *K18-hACE2*$^{+/-}$ **.** *P<0.05.)

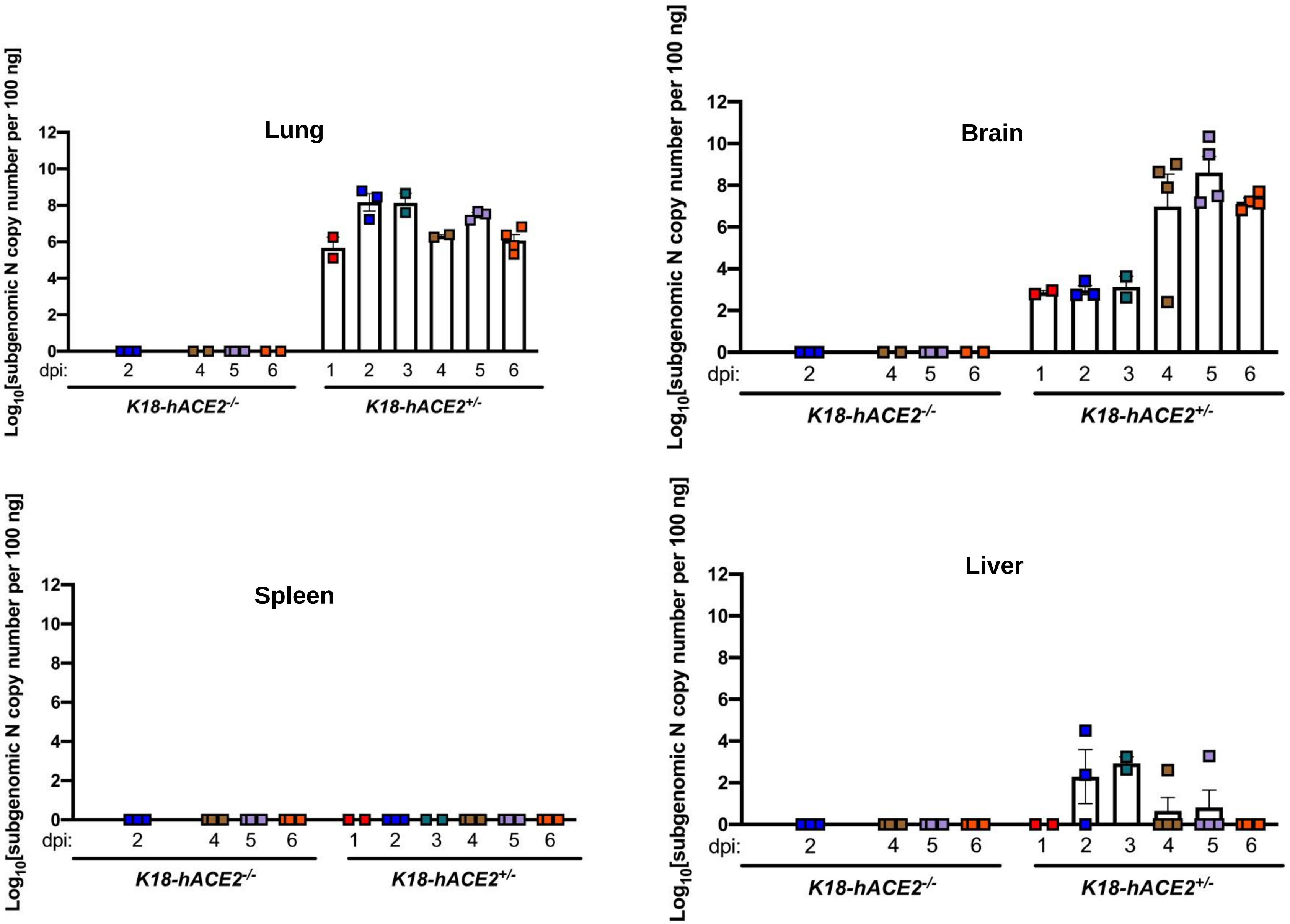

Figure S4. Daily viral subgenomic N mRNA (sgmRNA) levels in tissues of the infected mice. *K18-hACE2*$^{-/-}$ and *K18-hACE2*$^{+/-}$ were intranasally infected with SARS-CoV-2 (1 X 10^5) and euthanized at the different days post infection. The level of sgnmRNA in the various organs were measured by qRT-PCR.

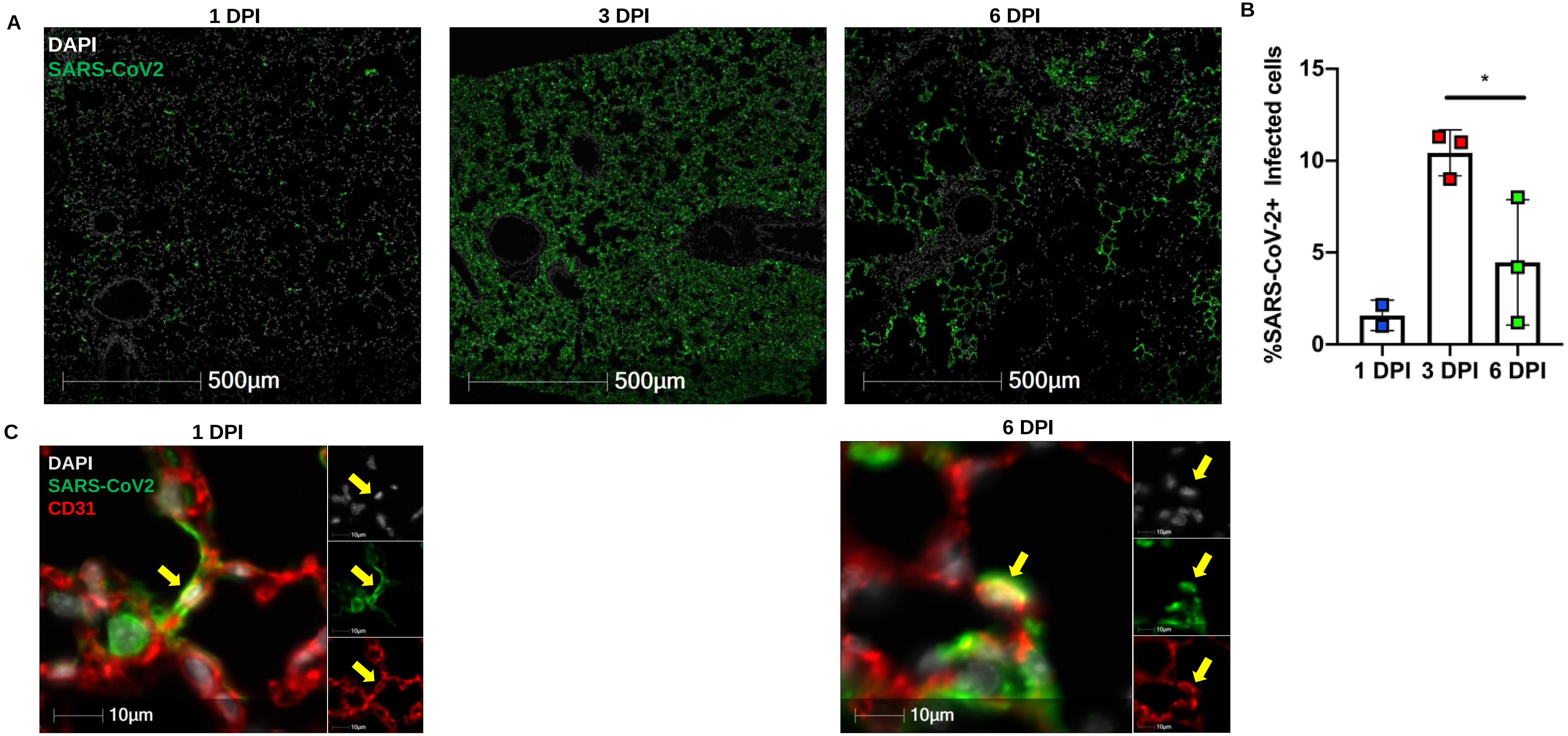

Figure S5. A, B. Immunostaining shows the viral stainings in the lungs of the infected K18-hACE2 mice at 1, 3, and 6 DPI. A. *K18-hACE2*$^{-/-}$ and *K18-hACE2*$^{+/-}$ were intranasally infected with SARS-CoV-2 (2 X 10^5) and were euthanized at the different day of the post infection assess. **B**. Quantification of virus infected cells were performed in the lungs of the infected K18-hACE2 mice. DPI: Day post infection. **C. Representative images show the co-staining of SARS-CoV-2 with CD31 in the lungs of the infected K18-hACE2 mice at 1 and 6 DPI (n=2)**.

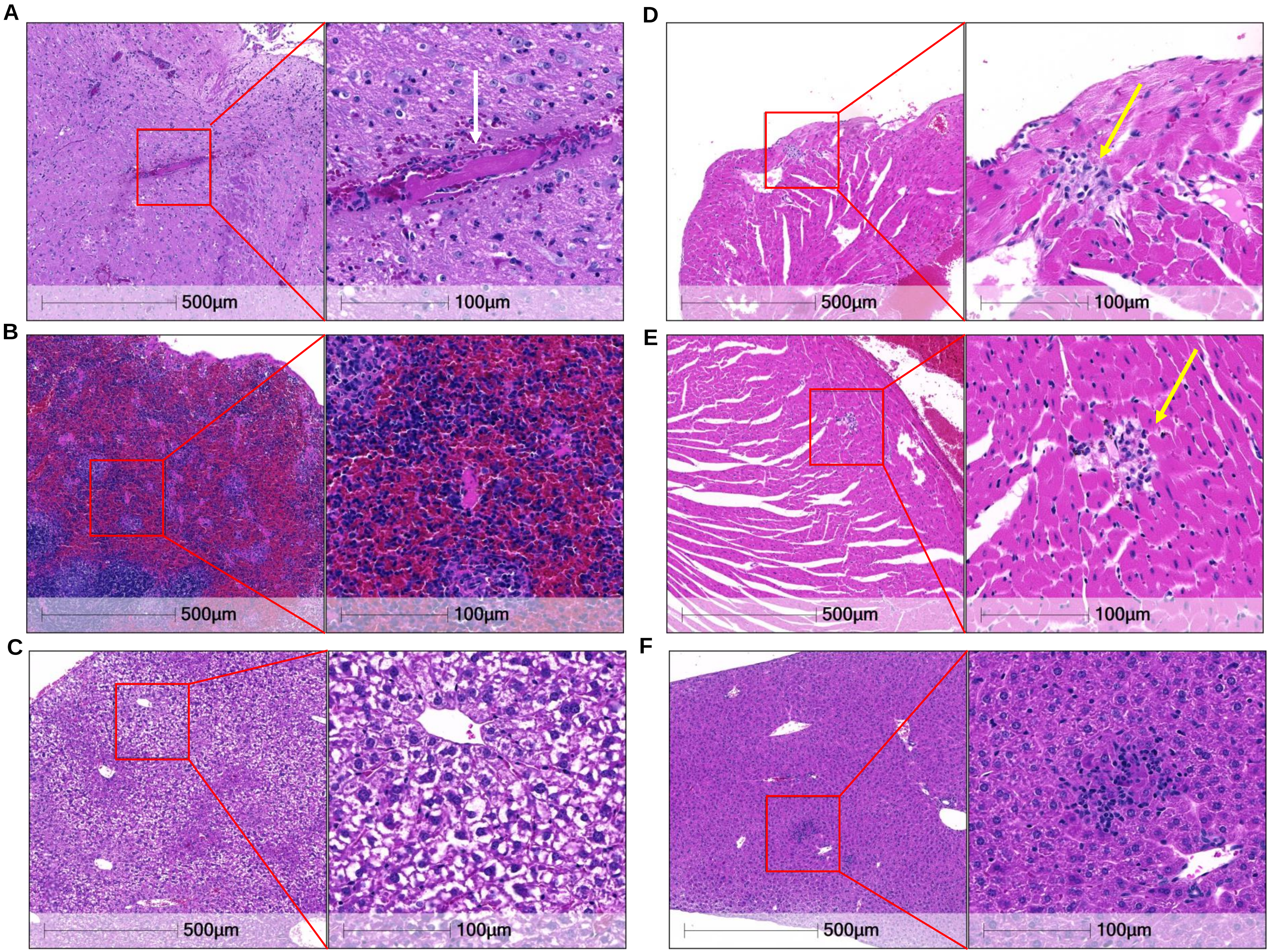

Figure S6: SARS-CoV-2 infection also mediated other organ damages in the K18-hACE2 mice at 7 DPI. 3 of 4 infected K18-hACE2 mice had other organ damages. **A, B and C**. One mouse had cerebral infarction with fibrin thrombi (white arrow)(**A**), splenic congestion (**B**), hepatocellular vacuolation (**C**) and minimal, focal myocarditis (yellow arrow) (**D**). **E**. Another mouse had focal myocarditis (pointed by yellow head arrow). **F.** The third mouse had a focal area of lymphocytic hepatitis.

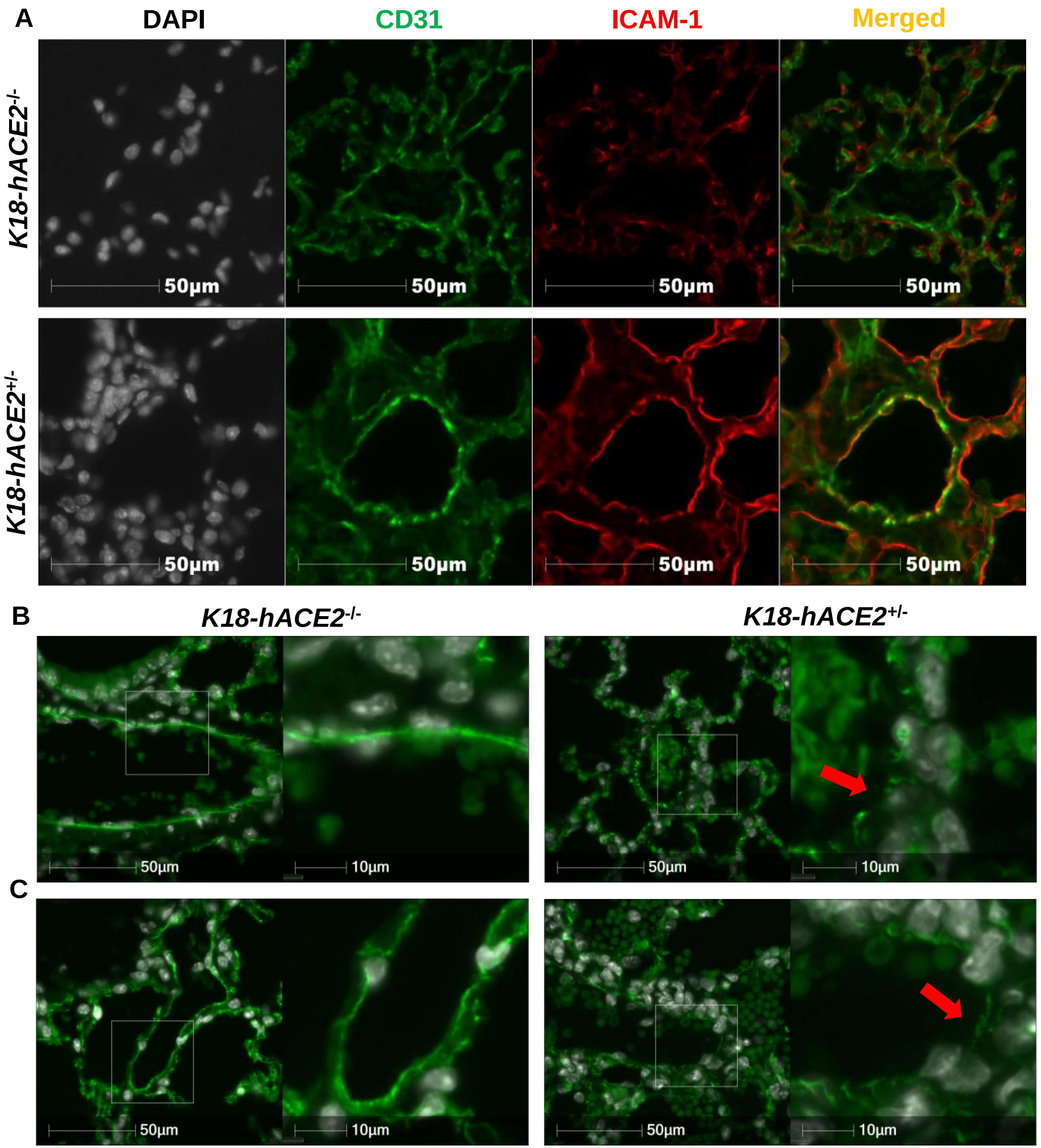

Figure S7:SARS-CoV-2 infection induces endothelial activation and dysfunction. (**A**) Co-staining of CD31 (green) and ICAM-1(red) in the lung sections from *K18*$^{-/-}$ (upper panel) and *K18*$^{+/-}$ mice (lower panel) at 6 DPI. (**B and C**) Representative image showing immmunofluorescence staining of VE-cadherin in the lung sections from two other infected *K18*$^{-/-}$ (left panel) and *K18*$^{+/-}$ mice (right panel) at 6 DPI, respectively. Red arrow points the changes of the endothelial integrity in the infected *K18*$^{+/-}$ mice.

A

B

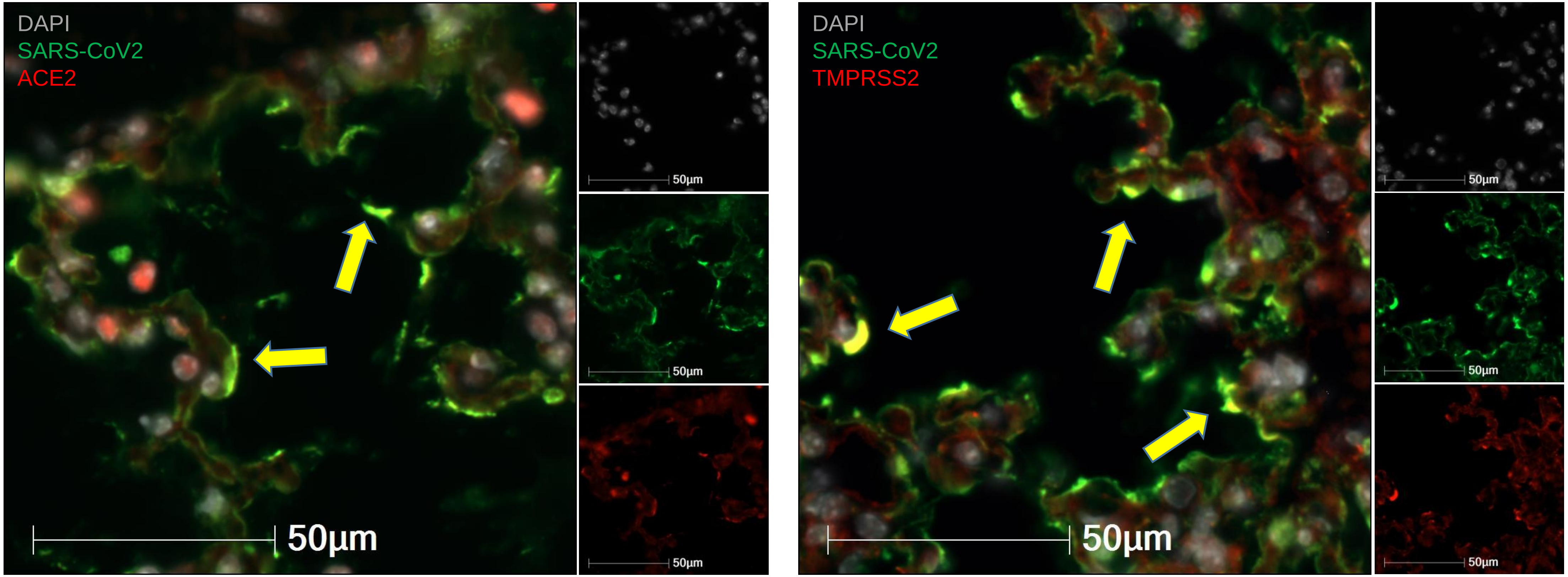

C

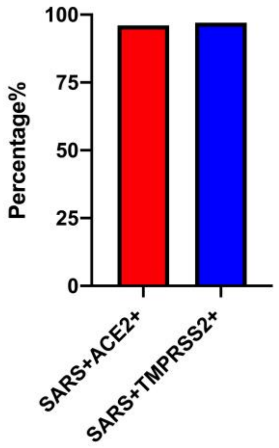

Figure S8. SARS-CoV-2 co-localizes with hACE2 or serine transmembrane protein 2 (Tmprss2). (A) Representative immunofluorescence staining of hACE2 (red) and SARS-CoV-2 (green) in lung sections from infected *K18*$^{+/-}$ at 3 DPI. Nuclei are stained with DAPI (white). Yellow arrows: co-localization of hACE2 and SARS-CoV-2 protein (N=3)**. (B)** Representative immunofluorescence staining of Tmprss2 (red) and SARS-CoV-2(green) in lung sections from infected *K18*$^{+/-}$ at 3 DPI. Nuclei are stained with DAPI (white). Yellow arrows: co-localization of Tmprss2 and SARS-CoV-2 protein (N=3). **(C)** Quantification of percentage of ACE2+ cells or TMPRSS2+ cells of total infected cells(SARS-CoV2+).

A

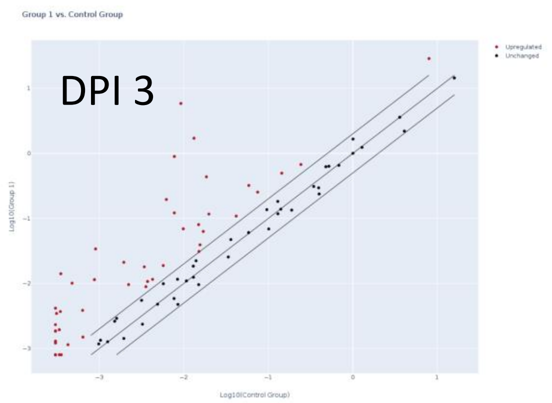

Gene Symbol	Fold Regulation	p-Value
Ccl19	3.44	0.003439
Cd70	4.27	0.040009
Csf1	3.40	0.035876
Csf3	4.00	0.000929
Cxcl10	633.29	0.042421
Ifna2	11.06	0.023155
Il15	2.60	0.030027
Il1a	2.52	0.022740
Il23a	2.68	0.012775
Il27	6.07	0.012313
Lta	5.77	0.011071

B

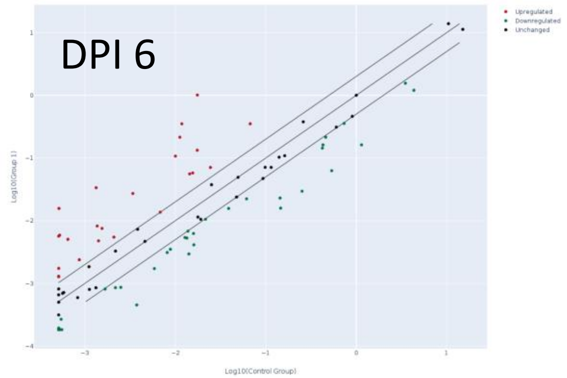

Gene Symbol	Fold Regulation	p-Value
Ccl12	7.62	0.002870
Ccl2	19.12	0.000000
Ccl3	3.71	0.008599
Ccl4	10.69	0.000160
Ccl5	5.22	0.000375
Ccl7	29.75	0.000000
Cxcl10	57.69	0.003025
Cxcl13	2.89	0.010333
Cxcl9	30.37	0.000002
Fasl	6.02	0.001725
Ifng	25.23	0.002745
Il10	11.04	0.007189
Il1rn	3.88	0.002453
Il6	11.16	0.000590
Xcl1	8.02	0.000112
Bmp2	-3.40	0.006106
Ccl17	-9.22	0.040496
Ctf1	-2.52	0.043312
Il7	-2.54	0.037074
Pf4	-6.26	0.021350
Ppbp	-8.54	0.018868

C

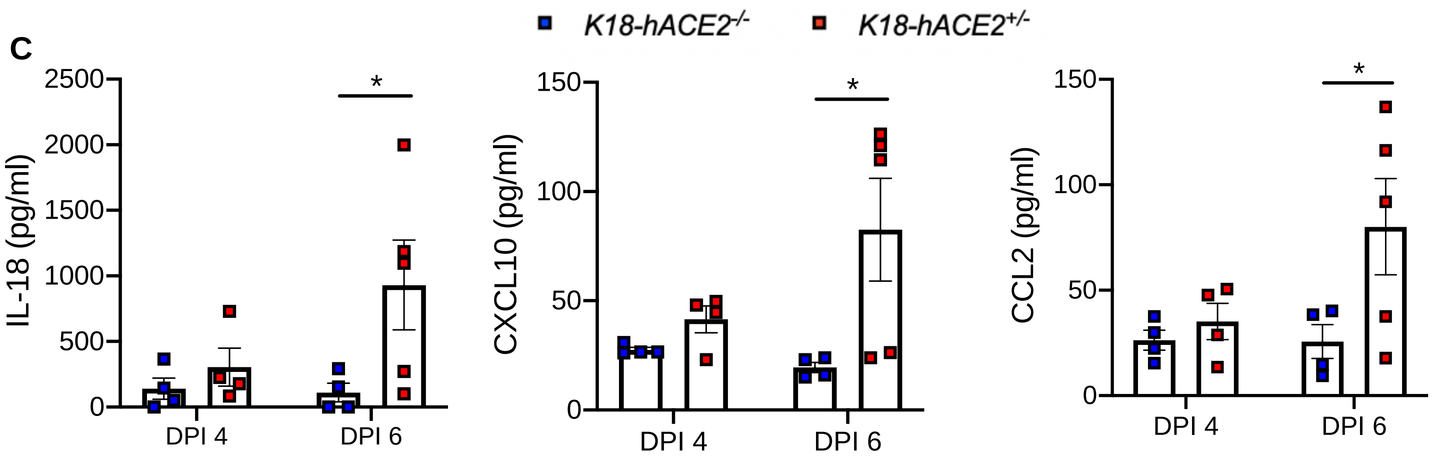

Figure S9. Cytokine levels in the lung and serum of infected K18-hACE2 mice. (**A,B**) The fold change of cytokines In the lung of infected K18-hACE2 compared to infected wild type mice at DPI 3 and 6. Scatter plot shown in left panel represents the relative transcript levels for each gene obtained from the infected *K18-hACE2*$^{+/-}$ mice plotted against the same gene from the infected *K18-hACE2*$^{-/-}$ mice. Genes with fold changes >2.0 are shown in the table on the right penal. (**C**) Serum IL-18, CXCL10 and CCL2 level in infected mice at DPI 4 and 6 is measured by multiplex method.

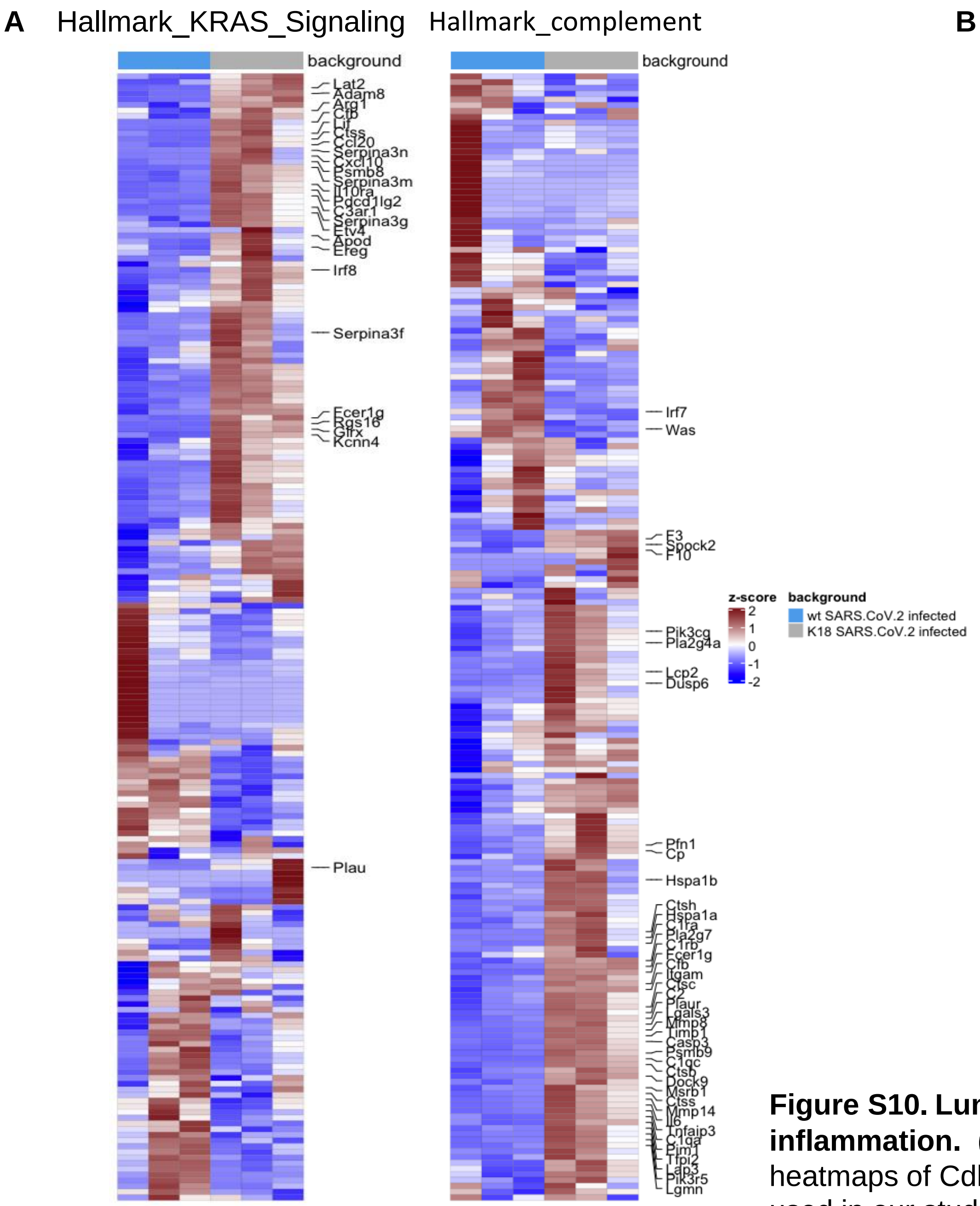

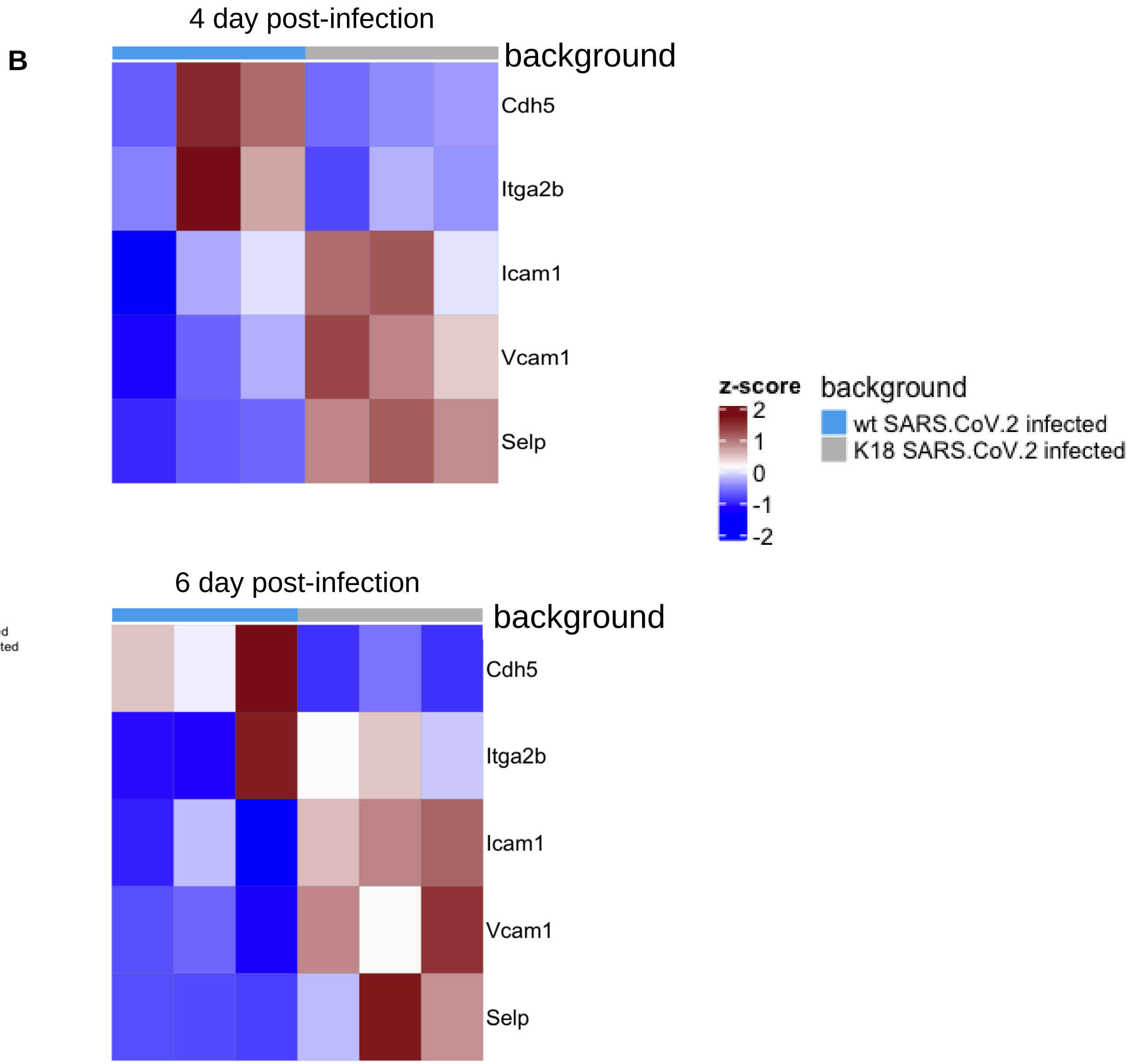

Figure S10. Lung tissue of SARS-CoV-2 infected K18 mice reveals gene signatures of endothelial inflammation. (A) Curated heatmaps of K-ras signaling and complement pathway.**(B)** Curated heatmaps of Cdh5 (or VE-cadherin), itga2b (or Cd41), Icam1, Vcam1, and Selp (P-selectin), biomarkers used in our studies for detecting EC dysfunction and platelet activation and aggregation.

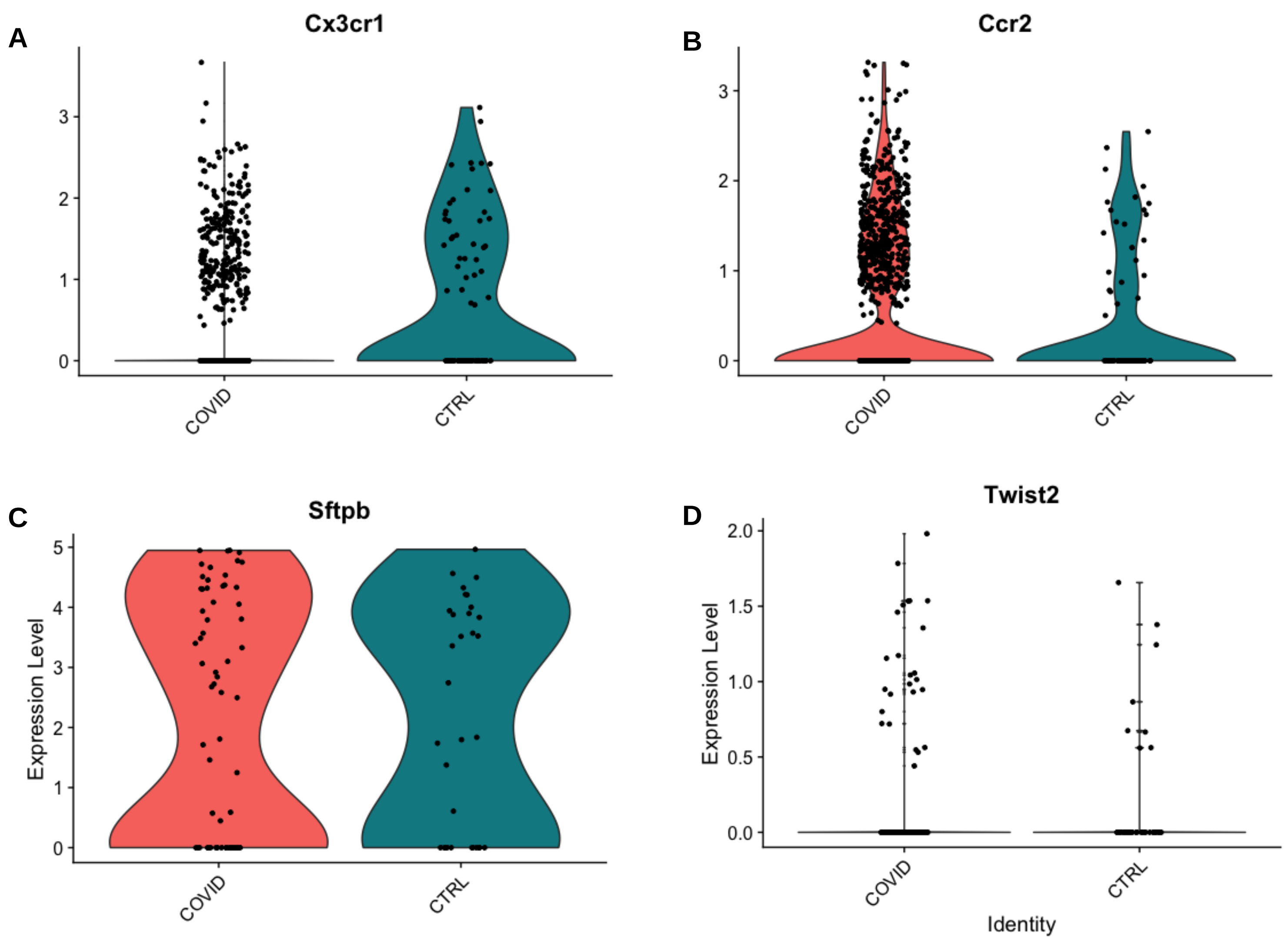

Figure S11. scRNAseq analysis/violin plots in the K18-ACE2 model. Frequencies of *Cx3cr1*and *Ccr2* macrophages in COVID as well as the type II pneumocyte gene *Sftpb* and the fibroblast markers *Twist2*.

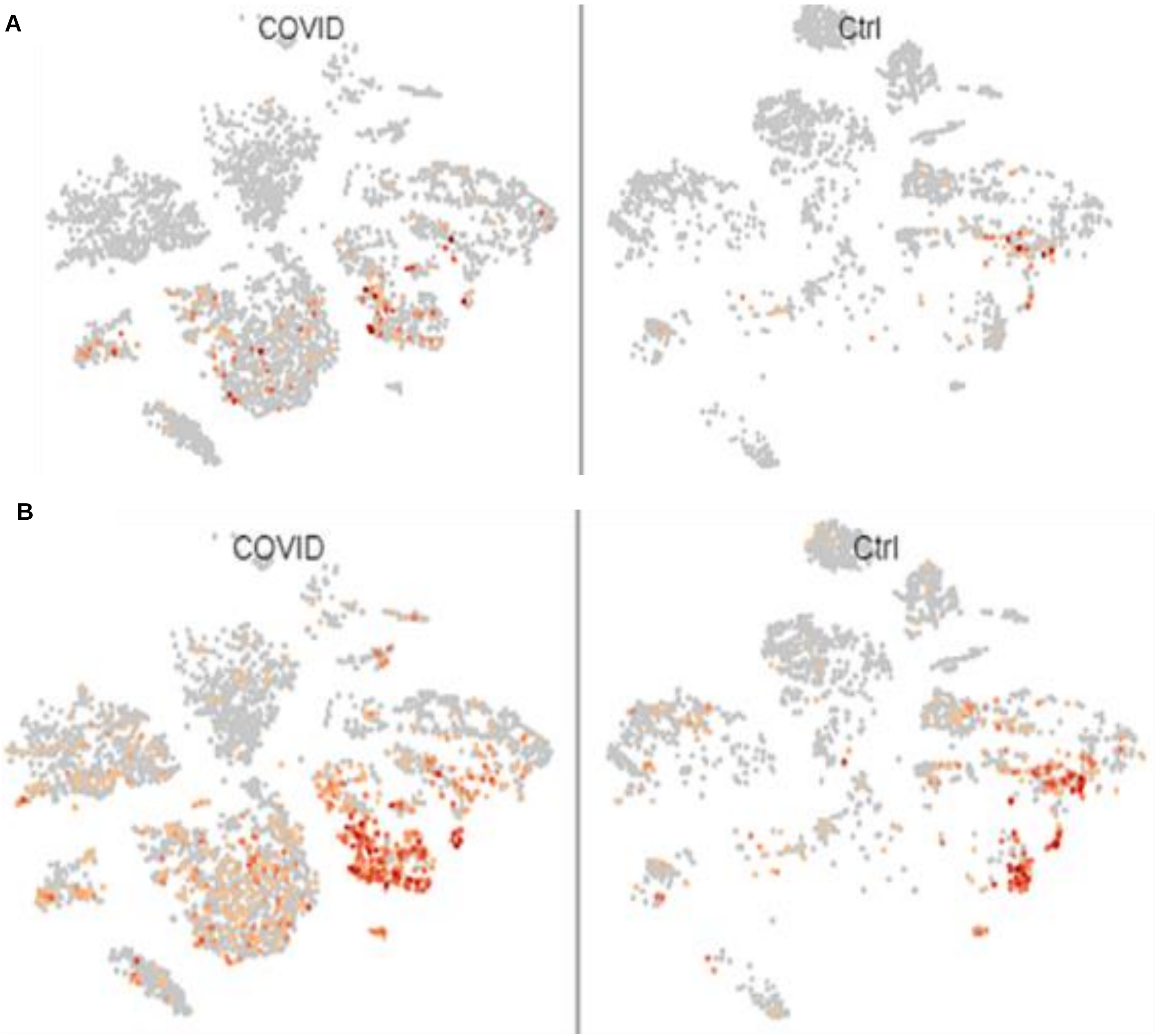

Figure S12. ***Cxcl9* and *Cxcl10* expression in infected *K18-hACE2* vis wild type mice. A.** *Cxcl9* expression**.** B. *Cxcl10* expression. COVID: Infected K18-hACE2. Ctrl: Infected wild type. COVID: Infected K18-hACE2; and Ctrl: Infected wild type.

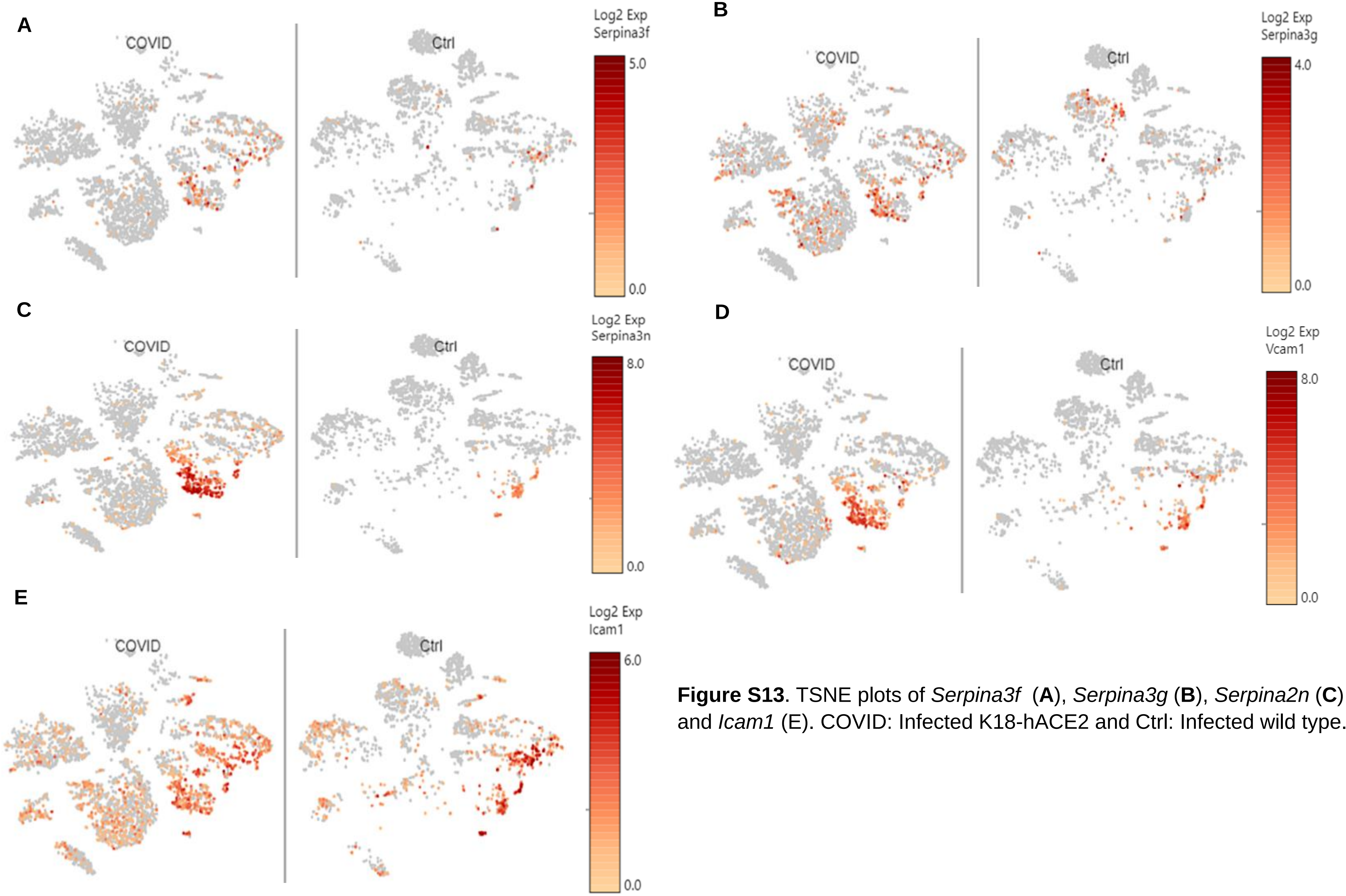

Figure S13. TSNE plots of *Serpina3f* (**A**), *Serpina3g* (**B**), *Serpina2n* (**C**), *Vcam1* (**D**) and *Icam1* (E). COVID: Infected K18-hACE2 and Ctrl: Infected wild type.

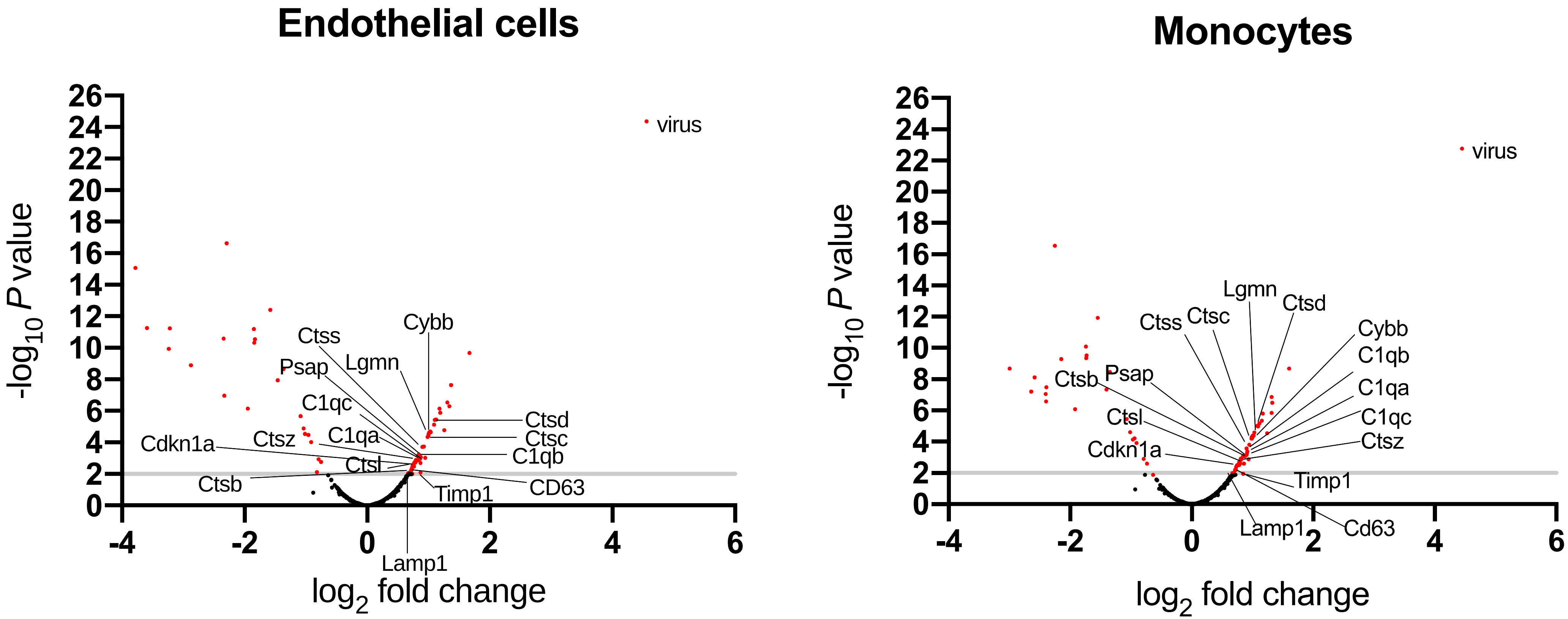

Figure S14 . Volcano plots of differentially expressed genes in virus+ and virus- endothelial cells and monocytes. To determine if specific pathways were regulated in specific cell clusters by the presence of viral RNA, we analyzed viral positive versus negative cells in the endothelial and monocyte cluster. In this analysis, we found upregulation of genes in the following pathways: Lysosome/Apoptosis: Ctsd, Lgmn, Ctsc, Ctss, Psap, Ctsb ,Ctsz, Ctsl, Cd63, Lamp1; HIF-1 signaling: Cybb, Timp1, Cdkn1a;and Complement: C1qb, C1qc, C1qa

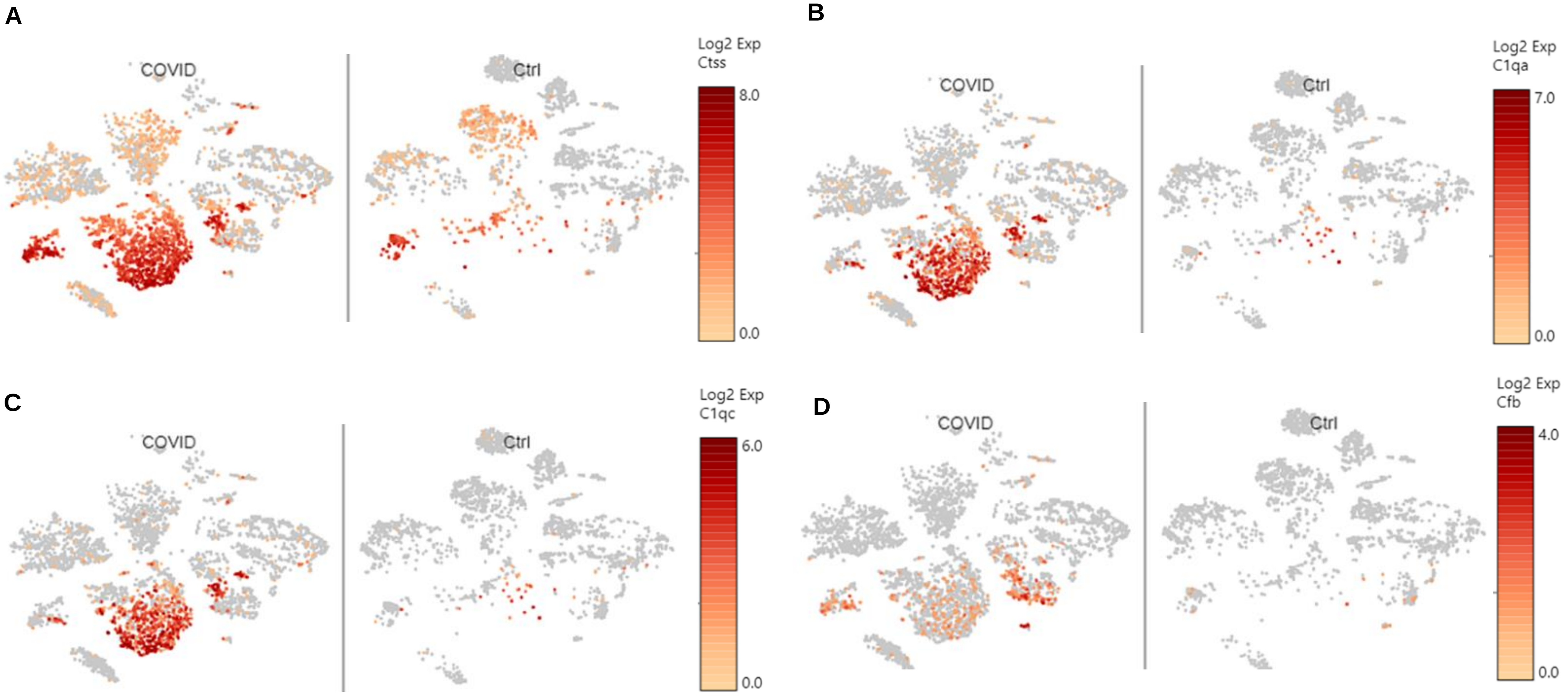

Figure S15. TSNE plots of *Ctss* (**A**) *C1qa* (**B**) *C1qb* (C) , and *Cfb* (D). COVID: Infected K18-hACE2; and Ctrl: Infected wild type.

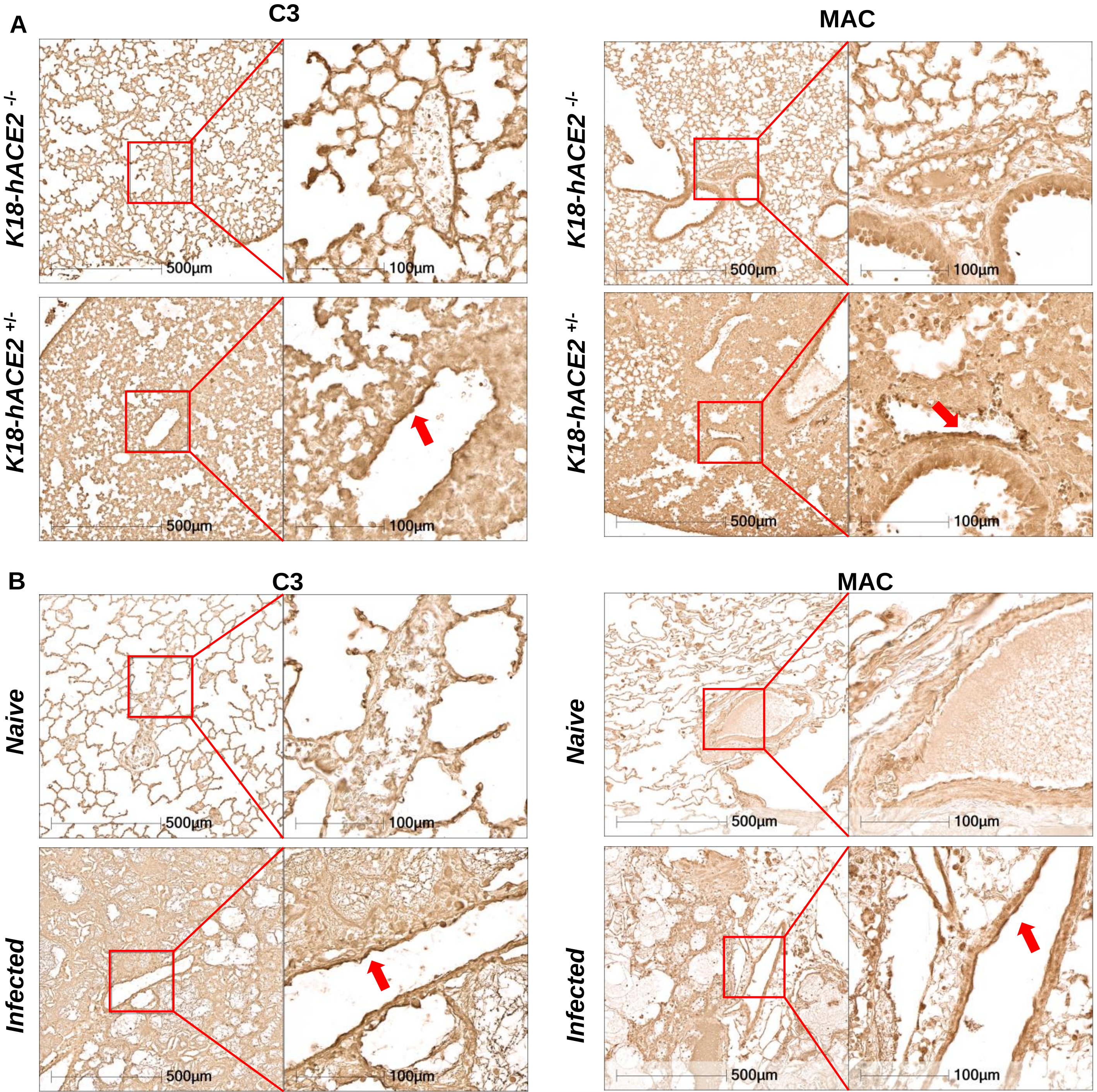

Figure S16.SARS-CoV-2 infection induces complement activation. A. Representative immunohistochemistry staining of C3 and C5b9 in lungs of SARS-CoV-2-infected *K18-hACE2*$^{-/-}$ and *K18-hACE2*$^{+/-}$ mice(n=3) showing increased C3 and MAC deposition in the pulmonary interstitial area and endothelial cells of SARS-CoV-2-infected *K18-hACE2*$^{-/-}$ (red arrow). **B.** Representative immunohistochemistry staining of C3 and C5b9 (MAC) in lungs of Naïve and SARS-CoV-2-infected African Green monkey (AGM) (n=3) showing increased C3 and MAC deposition in the pulmonary interstitial area and endothelial cells of SARS-CoV-2-infected AGMs(red arrow).

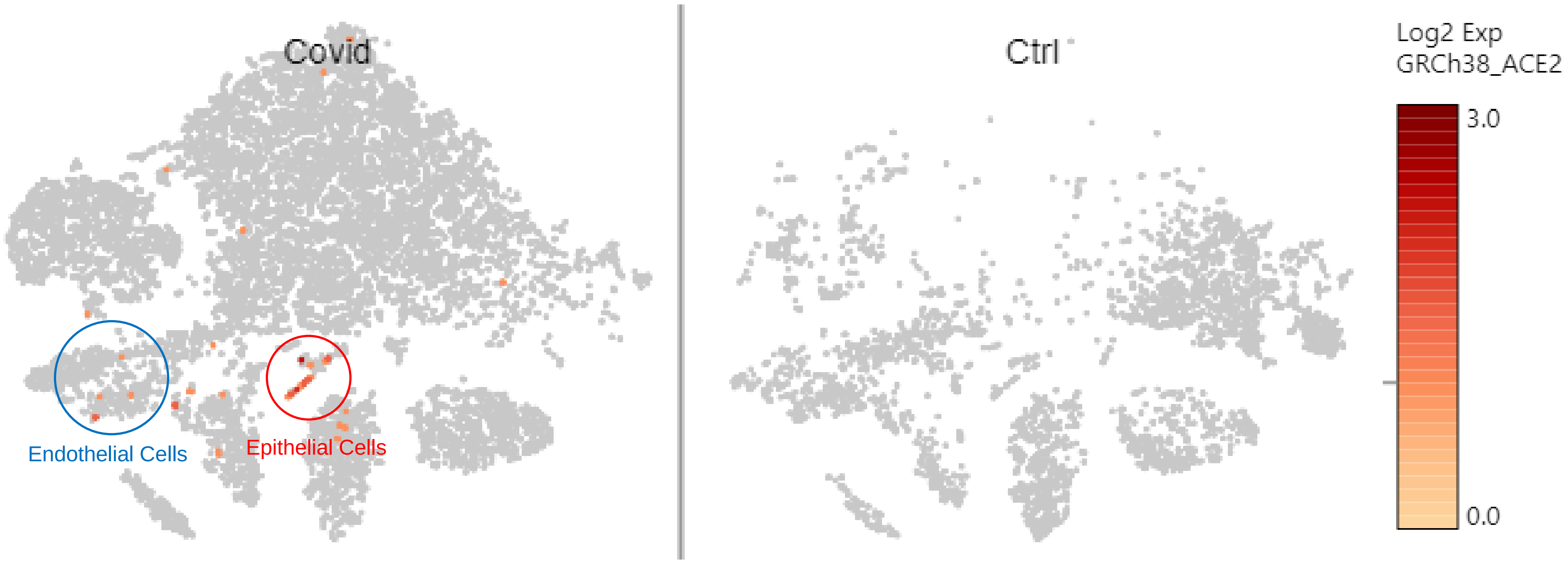

Figure S17. Human *ACE2* expression in SARS-CoV2 infected K18 transgenic mice. We identified ACE2 expression in both epithelial cells (including Club cells and Type II cells) as well as in the endothelial cells (left panel). No ACE2 expression was observed in SARS-CoV2 infected WT mice (right panel).

Parameters	*K18-hACE2*$^{-/-}$(N=5)	*K18-hACE2*$^{+/-}$ DPI 4(N=3)	T-test (P)	*K18-hACE2*$^{+/-}$ DPI 6(N=4)	T-test (P)
WBC,x10^3/ul	8.49±1.31	3.63±0.39	0.000546	5.08±2.52	0.065828
RBC,x10^6/ul	9.49±0.35	10.18±0.33	0.042902	11.70±0.57	0.001376
HGB,g/dL	13.96±0.46	14.53±0.45	0.152501	16.60±0.73	0.001661
HCT,%	45.80±2.61	44.33±1.85	0.392148	49.75±3.44	0.110791
MCV,fL	48.24±1.57	43.53±0.41	0.001594	42.53±1.13	0.0004
MCH,pg	14.70±0.1	14.27±0.06	0.000242	14.18±0.13	0.000612
MCHC,g/dL	30.54±0.91	32.80±0.36	0.003148	33.45±1.21	0.008706
RDW,%	16.80±0.29	17.20±0.17	0.051113	18.48±0.46	0.001549
PLTC,/uL	1686750±479967.6	2031000±19798.9	0.246964	1632000±733927.8	0.905341
MPV,FL	7.20±0.851	7.37±0.40	0.722036	6.95±0.13	0.55091
NEUT,%	6.34±0.974	7.37±2.02	0.479168	17.73±6.93	0.045062
LYMPH,%	86.18±3.71	78.97±10.54	0.357626	71.00±5.12	0.003487
MONO,%	4.70±2.94	11.07±9.92	0.382757	10.25±1.77	0.01093
EOS,%	2.34±0.71	1.43±1.71	0.459837	0.10±0.14	0.001598
BASO,%	0.44±0.18	1.17±0.25	0.01839	0.96±0.19	0.007067
NEUT, x10^3/ul	0.55±0.157	0.26±0.05	0.012497	0.83±0.29	0.148864
LYMPH, x10^3/ul	7.31±1.15	2.84±0.13	0.000833	3.67±1.97	0.024731
MONO, x10^3/ul	0.396±0.24	0.427±0.42	0.91603	0.538±0.31	0.48494
EOS, x10^3/ul	0.198±0.06	0.057±0.07	0.051177	0.005±0.005	0.001978
BASO, x10^3/ul	0.034±0.01	0.043±0.01	0.346339	0.045±0.017	0.339389

Table S1:CBC counting. P vs cell count in the infected-*K18-hACE2*$^{-/-}$ mice at 4 and 6 DPI.

Table S2

Anti-SARS(1:1000,NR-10361,BEI)

Anti-CD31(1:100,AF3628,R&D)

Anti-CD31(1:100M0823,DAKO)

Anti-VCAM-1(1:500,ab134047,Abcam)

Anti-ICAM-1(1:100,R&D,AF796),

Anti-VE-cadherin (1:100,R&D,AF1002) .

Anti- P-selectin (1:500, ab255822, Abcam),

CD41 (1:1000, ab134131, Abcam),

C5b-9 (1:200, ab55811, Abcam),

C3 (1:200, ab200999, Abcam)

Anti-hACE2(1:200, HPA000288,Sigma)

Anti-TMPRSS2(1:200, HPA035787,Sigma)
